# Regulation of *UHRF1* by dual-strand tumor-suppressor *microRNA-145* (*miR-145-5p* and *miR-145-3p*): inhibition of bladder cancer cell aggressiveness

**DOI:** 10.18632/oncotarget.8668

**Published:** 2016-04-09

**Authors:** Ryosuke Matsushita, Hirofumi Yoshino, Hideki Enokida, Yusuke Goto, Kazutaka Miyamoto, Masaya Yonemori, Satoru Inoguchi, Masayuki Nakagawa, Naohiko Seki

**Affiliations:** ^1^ Department of Urology, Graduate School of Medical and Dental Sciences, Kagoshima University, Kagoshima, Japan; ^2^ Department of Functional Genomics, Chiba University Graduate School of Medicine, Chuo-ku, Chiba, Japan

**Keywords:** miR-145-5p, miR-145-3p, tumor-suppressor, UHRF1, bladder cancer

## Abstract

In microRNA (miRNA) biogenesis, the guide-strand of miRNA integrates into the RNA induced silencing complex (RISC), whereas the passenger-strand is inactivated through degradation. Analysis of our miRNA expression signature of bladder cancer (BC) by deep-sequencing revealed that *microRNA* (*miR*)*-145-5p* (guide-strand) and *miR-145-3p* (passenger-strand) were significantly downregulated in BC tissues. It is well known that *miR-145-5p* functions as a tumor suppressor in several types of cancer. However, the impact of *miR-145-3p* on cancer cells is still ambiguous. The aim of the present study was to investigate the functional significance of *miR-145-3p* and BC oncogenic pathways and targets regulated by *miR-145-5p/miR-145-3p*. Ectopic expression of either *miR-145-5p* or *miR-145-3p* in BC cells significantly suppressed cancer cell growth, migration and invasion and it also induced apoptosis. The gene encoding ubiquitin-like with PHD and ring finger domains 1 (*UHRF1*) was a direct target of these miRNAs. Silencing of *UHRF1* induced apoptosis and inhibited cancer cell proliferation, migration, and invasion in BC cells. In addition, overexpressed *UHRF1* was confirmed in BC clinical specimens, and the high *UHRF1* expression group showed a significantly poorer cause specific survival rate in comparison with the low expression group. Taken together, our present data demonstrated that both strands of *miR-145* (*miR-145-5p*: guide-strand and *miR-145-3p*: passenger-strand) play pivotal roles in BC cells by regulating *UHRF1*. The identification of the molecular target of a tumor suppressive miRNAs provides novel insights into the potential mechanisms of BC oncogenesis and suggests novel therapeutic strategies.

## INTRODUCTION

In 2012, more than 400,000 new cases of bladder cancer (BC) were diagnosed and 165,000 patients died worldwide [1]. As for the prevalence of BC, men are three times more frequently diagnosed with BC than women [2]. The reasons for this disparity between sexes are not fully understood. BC is pathologically classified into two groups: non-muscle-invasive BC (NMIBC) and muscle-invasive BC (MIBC). Most BC patients (approximately 50%–80%) are diagnosed with NMIBC and this disease can be treated by removing the tumor by transurethral approaches [3]. In NMIBC, disease may recur, and some patients (approximately 25%) progress to MIBC [3]. Patients with advanced BC are generally treated with combination chemotherapy (gemcitabine and cisplatin), but progression-free survival is of limited duration [4]. Therefore, it is important to elucidate the molecular mechanisms of recurrence and invasiveness of BC cells to develop new treatment strategies.

The discovery of non-coding RNA in the human genome changed approaches in cancer research [5, 6]. Molecular mechanisms of post transcriptional gene regulation by protein-coding RNA/non-coding RNA networks are being studied on a genome-wide scale. MicroRNA (miRNA) is a class of small non-coding RNAs, and they are known to be involved in the repression or degradation of target RNA transcripts in a sequence-dependent manner [7]. A single miRNA can regulate thousands of target transcripts, and more than 60% of protein-coding genes may be influenced by miRNAs [8, 9]. Accumulating evidence indicates that aberrantly expressed miRNAs disturb normally regulated RNA networks, leading to pathologic responses in cancer cells [6]. Strategies to identify aberrant expression of miRNA-mediated cancer pathways are being developed as a new direction in cancer research in the post genome sequencing era.

To seek out differentially expressed miRNAs in BC cells, we used BC clinical specimens to establish deep sequencing-based miRNA expression signatures [10]. In general, the guide-strand RNA from duplex miRNA is retained to direct recruitment of the RNA induced silencing complex (RISC) to target messenger RNAs, whereas the passenger-strand RNA is degraded [11–13]. Recently, we revealed that both strands of *microRNA* (*miR*)*-144-5p* and *miR-144-3p* derived from *pre-miR-144* acted as tumor suppressors in BC cells [14]. Moreover, *miR-144-5p* (passenger-strand) directly targeted *cyclin E1* and *E2* in BC cells, suggesting that the passenger-strand of miRNA has a physiological role in cells [14].

In this study, we focused on *miR-145-5p* and *miR-145-3p* because these miRNAs were significantly downregulated in BC cells as determined in our deep sequencing signature [10]. It is well known that *miR- 145- 5p* functions as a tumor suppressor in several types of cancer, including BC [15]. However, the role of *miR-145-3p* on cancer cells is still ambiguous. The aims of the present study were to investigate the anti-tumor effects of *miR-145-3p* as well as *miR-145-5p*, and to determine the BC oncogenic pathways and target genes regulated by these miRNAs. The discovery that *miR- 145- 5p* and *miR-145-3p* coordinately regulate pathways and targets provides new insight into the mechanisms of BC progression and metastasis.

## RESULTS

### The expression levels of *miR-145-5p* and *miR-145-3p* in BC specimens and cell lines

**Figure 1:**
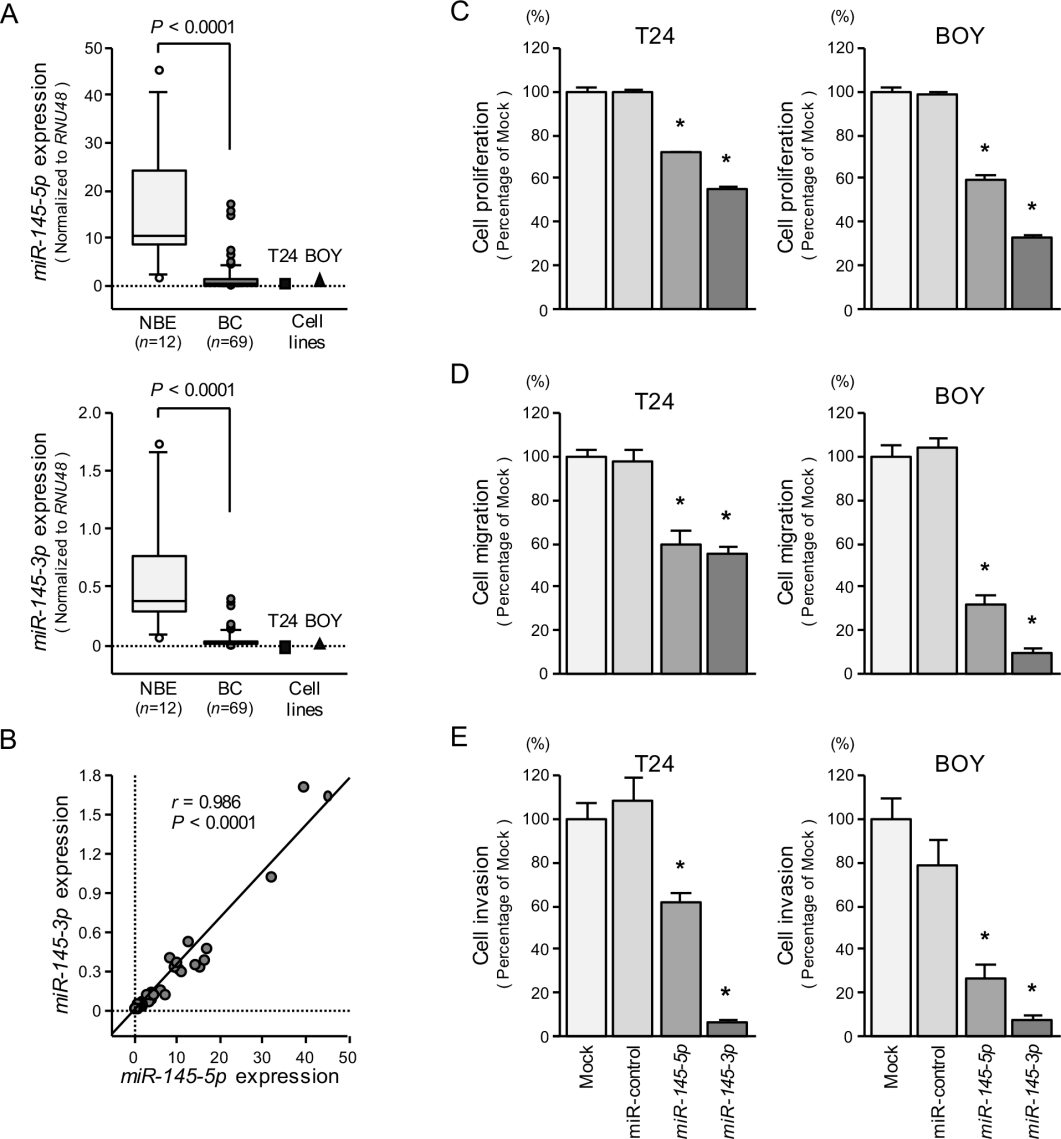
The expression levels of *miR-145-5p* and *miR-145-3p*, and their effects in BC cells (**A**) Expression levels of *miR- 145- 5p* and *miR-145-3p* in clinical specimens and BC cell lines were determined by qRT-PCR. Data were normalized to *RNU48* expression. (**B**) Correlation of *miR-145-5p* and *miR-145-3p* expression. (**C**) Cell growth was determined by XTT assays 72 hours after transfection with 10 nM *miR-145-5p* or *miR-145-3p*. *P < 0.0001. (**D**) Cell migration activity was determined by the wound-healing assays. *P < 0.0001. (**E**) Cell invasion activity was determined using Matrigel invasion assays. *P < 0.0001.

We evaluated the expression levels of *miR-145-5p* and *miR-145-3p* in BC tissues (*n* = 69), normal bladder epithelia (NBE) (*n* = 12), and two BC cell lines (T24 and BOY). The expression levels of *miR-145-5p* and *miR- 145- 3p* were significantly lower in tumor tissues and BC cell lines compared with NBE (Figure 1A). Spearman's rank test showed a positive correlation between the expression of these miRNAs (*r* = 0.986 and *P* < 0.0001) (Figure 1B). On the other hand, there were no significant relationships between any of the clinicopathological parameters (i.e., tumor grade, stage, metastasis, or survival rate) and the expression levels of *miR-145-5p* and *miR-145-3p* (data not shown).

### Effect of restoring *miR-145-5p* or *miR-145- 3p* expression on cell growth, migration, and invasion in BC cell lines

We performed gain-of-function studies using transfection of these miRNAs to investigate their functional roles. XTT, cell migration, and invasion assays demonstrated that cell proliferation, cell migration, and cell invasion were significantly inhibited in *miR-145-5p* and *miR-145-3p* transfectants in comparison with mock or miR-control transfectants (each *P* < 0.0001, Figure 1C, 1D, and 1E). These results suggested that *miR-145-3p* as well as *miR-145-5p* could have a tumor suppressive function in BC cells.

To investigate the synergistic effects of *miR- 145- 5p* and *miR-145-3p*, we performed proliferation, migration, and invasion assays with co-transfection of *miR- 145-5p* and *miR-145-3p* in BC cells (T24 and BOY), but they did not show synergistic effects of these miRNAs transfection (Supplementary Figure 1).

### Effects of *miR-145-5p* and *miR-145-3p* transfection on apoptosis and cell cycle in BC cell lines

Because *miR-145-5p* and *miR-145-3p* transfection strongly inhibited cell proliferation in BC cell lines, we hypothesized that these miRNAs may induce apoptosis. Hence, we performed flow cytometric analyses to determine the number of apoptotic cells following restoration of *miR- 145-5p* or *miR-145-3p* expression.

**Figure 2:**
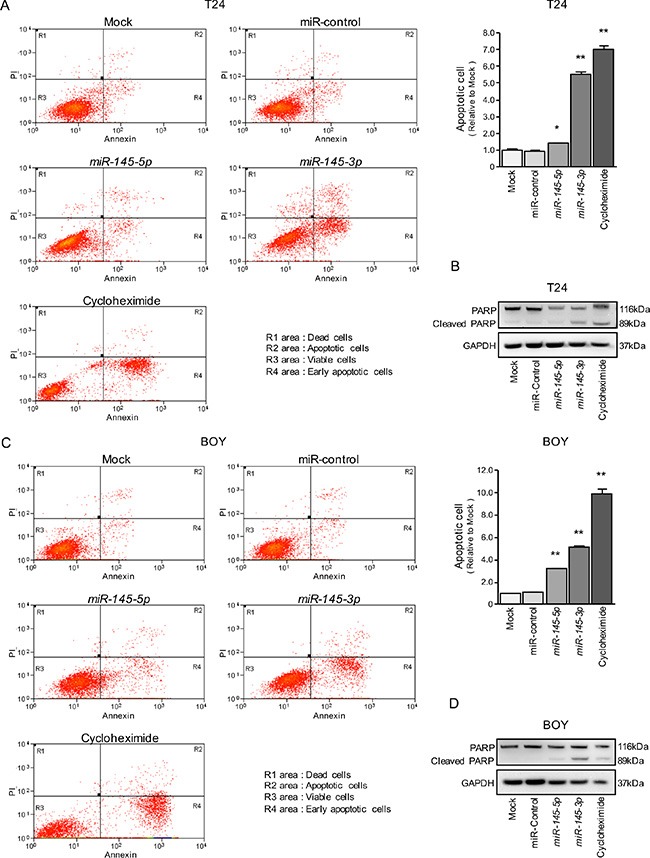
Effects of *miR-145-5p* and *miR-145-3p* on apoptosis (**A**, **C**) Apoptosis assays were carried out using flow cytometry. Early apoptotic cells are in area R4 and apoptotic cells are in area R2. The normalized ratios of apoptotic cells are shown in the histograms. Cycloheximide (2 μg/mL) was used as positive control. *P = 0.0266 and **P < 0.0001. (**B**, **D**) Western blot analyses for apoptotic markers (cleaved PARP) in BC cell lines. GAPDH was used as a loading control.

The apoptotic cell numbers (apoptotic and early apoptotic cells) were significantly larger in *miR-145-5p* or *miR-145-3p* transfectants than in mock or miR-control transfectants (Figure 2A and 2C). Western blot analyses showed that cleaved PARP expression was significantly increased in *miR-145-5p* or *miR-145-3p* transfectants compared with mock or miR-control transfectants (Figure 2B and 2D).

We also investigated the cell cycle assays using *miR-145-5p* and *miR-145-3p* transfectants. The fraction of cells in the G2/M phase was significantly larger in *miR-145-5p* and *miR-145-3p* transfectants in T24 cells in comparison with mock or miR-control transfectants (Supplementary Figure 2). In contrast, *miR-145-5p* and *miR-145-3p* transfection induced cell cycle arrest at the G1 phase in BOY cells (Supplementary Figure 2). The reason why the cell cycle arrest (G2 arrest in T24 and G1 arrest in BOY) varies according to a cell types is a future problem.

### Identification of common target genes regulated by *miR-145-5p* and *miR-145-3p* in BC cells

**Figure 3:**
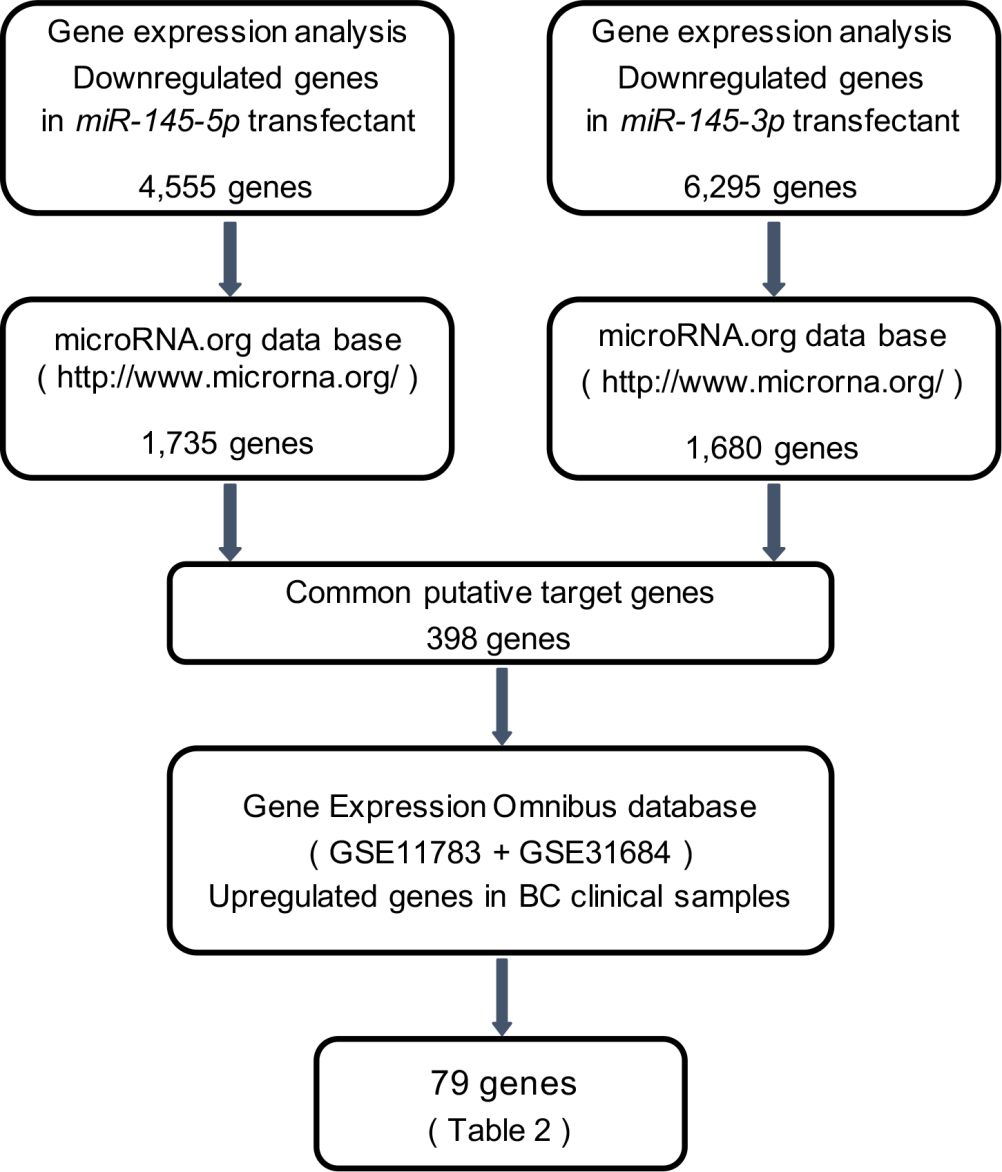
Flow chart illustrates the strategy for analysis of *miR-145-5p* and *miR-145-3p* target genes A total of 4,555 and 6,295 downregulated genes in expression analysis of *miR-145-5p* and *miR-145-3p* transfected BC cell lines, respectively, (T24 and BOY) were selected as putative target genes. Next we merged the data of those selected genes and the microRNA.org database. The analyses showed 398 common putative target genes between *miR-145-5p* and *miR-145-3p*. We then analyzed gene expression with available GEO data sets (GSE11783 + GSE31684). The analyses showed that 79 genes were significantly upregulated in BC specimens compared with NBE.

To gain further insight into the molecular mechanisms and pathways regulated by tumor suppressive *miR-145-5p* and *miR-145-3p* in BC cells, we used a combination of *in silico* analyses and gene expression analyses. Figure 3 shows our strategy to narrow down the common target genes of *miR-145-5p* and *miR-145-3p*.

In gene expression analyses, a total of 4,555 and 6,295 genes were downregulated in *miR-145-5p* and *miR- 145-3p* transfectants, respectively, in comparison with control transfectants (Gene Expression Omnibus (GEO), accession number: GSE66498). Of those downregulated genes, 1,735 and 1,680 genes, respectively, had putative binding sites for *miR-145-5p* and *miR-145- 3p* in their 3′ untranslated regions (UTRs) according to the microRNA.org database. We found that there were 398 common genes targeted by both miRNAs, and among them, we ultimately identified 79 genes that were upregulated in the clinical BC samples from the GEO (accession numbers: GSE11783, GSE31684) (Table 1). We subsequently focused on the ubiquitin-like with PHD and ring finger domains 1 (*UHRF1*) gene because it was the top ranked gene in the list.

**Table 1:** Highly expressed genes putatively regulated by *miR-145-5p* and *miR-145-3p*

Entrez Gene ID	Gene Symbol	Description	Genomic location	Gene Expression Omnibus (GSE11783 + GSE31684)			Expression in *miR-145-5p*transfectant (Log_2_ FC)		Expression in *miR-145-3p*transfectant (Log_2_ FC)	
				Expression	Log_2_ FC	*P*-value	T24	BOY	T24	BOY
29128	*UHRF1*	ubiquitin-like with PHD and ring finger domains 1	19p13.3	up	4.984	1.049E-03	−0.041	−0.274	−0.334	−0.901
54972	*TMEM132A*	transmembrane protein 132A	11q12.2	up	3.458	1.049E-03	−0.006	−0.087	−0.178	−0.140
4288	*MKI67*	marker of proliferation Ki-67	10q26.2	up	3.182	1.049E-03	−0.070	−0.022	−0.609	−0.872
1111	*CHEK1*	checkpoint kinase 1	11q24.2	up	2.841	1.049E-03	−0.354	−0.204	−0.426	−0.583
25886	*POC1A*	POC1 centriolar protein A	3p21.2	up	2.354	1.049E-03	−0.146	−0.194	−0.251	−0.161
400745	*SH2D5*	SH2 domain containing 5	1p36.12	up	2.299	1.049E-03	−0.512	−0.075	−0.136	−0.038
55215	*FANCI*	Fanconi anemia, complementation group I	15q26.1	up	2.188	1.049E-03	−0.031	−0.079	−0.281	−0.320
51512	*GTSE1*	G-2 and S-phase expressed 1	22q13.31	up	2.147	1.049E-03	−0.028	−0.149	−0.713	−0.209
157570	*ESCO2*	establishment of sister chromatid cohesion N-acetyltransferase 2	8p21.1	up	2.028	1.049E-03	−0.441	−0.352	−0.585	−0.166
2175	*FANCA*	Fanconi anemia, complementation group A	16q24.3	up	1.877	1.049E-03	−0.017	−0.166	−0.412	−0.532
6624	*FSCN1*	fascin homolog 1, actin-bundling protein (Strongylocentrotus purpuratus)	7p22.1	up	1.829	2.942E-03	−2.899	−0.732	−0.175	−1.133
22979	*EFR3B*	EFR3 homolog B (S. cerevisiae)	2p23.3	up	1.803	1.247E-03	−0.312	−0.033	−1.189	−1.625
3918	*LAMC2*	laminin, gamma 2	1q25.3	up	1.797	1.791E-02	−0.839	−0.707	−0.125	−0.608
8349	*HIST2H2BE*	histone cluster 2, H2be	1q21.2	up	1.764	1.524E-03	−0.266	−0.149	−0.524	−0.170
9455	*HOMER2*	homer homolog 2 (Drosophila)	15q25.2	up	1.706	2.526E-03	−0.360	−0.278	−0.132	−0.305
25902	*MTHFD1L*	methylenetetrahydrofolate dehydrogenase (NADP+ dependent) 1-like	6q25.1	up	1.611	1.049E-03	−0.307	−0.024	−0.617	−0.505
55732	*C1orf112*	chromosome 1 open reading frame 112	1q24.2	up	1.461	1.685E-03	−0.099	−0.147	−0.030	−0.132
388389	*CCDC103*	coiled-coil domain containing 103	17q21.31	up	1.390	3.290E-02	−0.327	−0.266	−2.471	−1.838
6566	*SLC16A1*	solute carrier family 16 (monocarboxylate transporter), member 1	1p13.2	up	1.359	3.893E-02	−0.229	−0.137	−0.759	−1.259
23178	*PASK*	PAS domain containing serine/threonine kinase	2q37.3	up	1.333	1.058E-03	−0.016	−0.001	−0.218	−0.443
5426	*POLE*	polymerase (DNA directed), epsilon, catalytic subunit	12q24.33	up	1.241	1.247E-03	−0.094	−0.424	−0.295	−0.051
55379	*LRRC59*	leucine rich repeat containing 59	17q21.33	up	1.233	1.049E-03	−0.155	−0.198	−0.289	−0.283
6715	*SRD5A1*	steroid-5-alpha-reductase, alpha polypeptide 1 (3-oxo-5 alpha-steroid delta 4-dehydrogenase alpha 1)	5p15.31	up	1.170	5.069E-03	−0.329	−0.018	−0.823	−0.837
4602	*MYB*	v-myb avian myeloblastosis viral oncogene homolog	6q23.3	up	1.160	4.501E-03	−0.105	−0.337	−0.111	−1.418
8940	*TOP3B*	topoisomerase (DNA) III beta	22q11.22	up	1.157	9.078E-03	−0.108	−0.021	−0.840	−1.150
64768	*IPPK*	inositol 1,3,4,5,6-pentakisphosphate 2-kinase	9q22.31	up	1.153	1.072E-03	−0.526	−0.102	−0.630	−0.296
9266	*CYTH2*	cytohesin 2	19q13.33	up	1.127	1.049E-03	−0.226	−0.104	−0.598	−0.377
221468	*TMEM217*	transmembrane protein 217	6p21.2	up	1.081	4.734E-02	−0.049	−0.008	−0.033	−0.337
25859	*PART1*	prostate androgen-regulated transcript 1 (non-protein coding)	5q12.1	up	1.025	4.873E-03	−0.144	−0.212	−0.097	−0.694
8566	*PDXK*	pyridoxal (pyridoxine, vitamin B6) kinase	21q22.3	up	1.014	1.316E-03	−0.039	−0.842	−0.567	−0.558
11072	*DUSP14*	dual specificity phosphatase 14	17q12	up	1.008	2.440E-03	−0.126	−0.092	−0.924	−1.020
23516	*SLC39A14*	solute carrier family 39 (zinc transporter), member 14	8p21.3	up	0.999	3.435E-03	−0.540	−0.216	−2.083	−1.548
85414	*SLC45A3*	solute carrier family 45, member 3	1q32.1	up	0.977	3.435E-03	−0.578	−0.086	−0.782	−0.505
1163	*CKS1B*	CDC28 protein kinase regulatory subunit 1B	1q21.3	up	0.941	1.857E-02	−0.370	−0.229	−0.678	−0.802
79929	*MAP6D1*	MAP6 domain containing 1	3q27.1	up	0.927	1.093E-03	−0.135	−0.210	−0.928	−0.529
65985	*AACS*	acetoacetyl-CoA synthetase	12q24.31	up	0.919	1.058E-03	−0.555	−0.367	−0.816	−0.798
1263	*PLK3*	polo-like kinase 3	1p34.1	up	0.910	1.685E-03	−0.229	−0.092	−1.766	−2.103
64785	*GINS3*	GINS complex subunit 3 (Psf3 homolog)	16q21	up	0.891	1.740E-03	−0.185	−0.218	−0.853	−0.826
4957	*ODF2*	outer dense fiber of sperm tails 2	9q34.11	up	0.854	1.185E-03	−0.232	−0.409	−0.610	−0.963
57613	*KIAA1467*	KIAA1467	12p13.1	up	0.837	4.169E-03	−0.382	−0.282	−0.398	−0.456
7525	*YES1*	v-yes-1 Yamaguchi sarcoma viral oncogene homolog 1	18p11.32	up	0.794	2.526E-03	−0.382	−0.447	−0.256	−0.446
8751	*ADAM15*	ADAM metallopeptidase domain 15	1q22	up	0.787	6.433E-03	−0.233	−0.217	−0.383	−0.318
7172	*TPMT*	thiopurine S-methyltransferase	6p22.3	up	0.786	1.524E-03	−0.167	−0.032	−0.482	−0.323
4615	*MYD88*	myeloid differentiation primary response 88	3p22.2	up	0.759	1.947E-03	−0.662	−0.118	−0.286	−0.113
1678	*TIMM8A*	translocase of inner mitochondrial membrane 8 homolog A (yeast)	Xq22.1	up	0.729	2.723E-03	−0.530	−0.187	−0.201	−0.267
3927	*LASP1*	LIM and SH3 protein 1	17q12	up	0.692	2.348E-03	−0.280	−0.014	−0.319	−0.069
10295	*BCKDK*	branched chain ketoacid dehydrogenase kinase	16p11.2	up	0.685	6.186E-03	−0.281	−0.161	−0.439	−0.246
26088	*GGA1*	golgi-associated, gamma adaptin ear containing, ARF binding protein 1	22q13.1	up	0.668	1.049E-03	−0.010	−0.074	−0.180	−0.202
6240	*RRM1*	ribonucleotide reductase M1	11p15.4	up	0.667	4.582E-02	−0.206	−0.207	−1.158	−2.292
219902	*TMEM136*	transmembrane protein 136	11q23.3	up	0.667	3.574E-03	−0.449	−0.477	−0.386	−0.405
7019	*TFAM*	transcription factor A, mitochondrial	10q21.1	up	0.644	1.274E-02	−0.163	−0.413	−0.543	−0.609
55775	*TDP1*	tyrosyl-DNA phosphodiesterase 1	14q32.11	up	0.624	1.316E-03	−0.151	−0.193	−0.651	−0.188
79858	*NEK11*	NIMA-related kinase 11	3q22.1	up	0.613	1.626E-03	−0.628	−0.563	−0.179	−0.189
1889	*ECE1*	endothelin converting enzyme 1	1p36.12	up	0.604	3.635E-02	−0.949	−0.274	−0.559	−0.639
65264	*UBE2Z*	ubiquitin-conjugating enzyme E2Z	17q21.32	up	0.590	1.348E-03	−0.352	−0.187	−0.895	−1.241
9205	*ZMYM5*	zinc finger, MYM-type 5	13q12.11	up	0.582	7.805E-03	−0.413	−0.381	−0.699	−0.890
996	*CDC27*	cell division cycle 27	17q21.32	up	0.572	9.799E-03	−0.486	−0.018	−0.260	−0.099
22898	*DENND3*	DENN/MADD domain containing 3	8q24.3	up	0.570	1.016E-02	−0.235	−0.012	−0.597	−0.926
84314	*TMEM107*	transmembrane protein 107	17p13.1	up	0.570	2.965E-02	−0.471	−0.208	−0.199	−0.839
85464	*SSH2*	slingshot protein phosphatase 2	17q11.2	up	0.562	2.440E-03	−0.296	−0.173	−0.433	−0.220
56180	*MOSPD1*	motile sperm domain containing 1	Xq26.3	up	0.559	1.928E-02	−0.145	−0.237	−1.352	−1.270
6625	*SNRNP70*	small nuclear ribonucleoprotein 70kDa (U1)	19q13.33	up	0.554	1.725E-02	−0.373	−0.281	−0.663	−0.988
60490	*PPCDC*	phosphopantothenoyl­- cysteine decarboxylase	15q24.2	up	0.550	1.182E-02	−0.269	−0.338	−0.057	−0.130
147657	*ZNF480*	zinc finger protein 480	19q13.41	up	0.547	3.893E-02	−0.453	−0.035	−0.107	−0.047
159090	*FAM122B*	family with sequence similarity 122B	Xq26.3	up	0.543	2.865E-02	−0.356	−0.131	−1.379	−1.493
3150	*HMGN1*	high mobility group nucleosome binding domain 1	21q22.2	up	0.522	7.521E-03	−0.884	−0.157	−0.162	−0.119
7421	*VDR*	vitamin D (1,25-dihydroxyvitamin D3) receptor	12q13.11	up	0.494	3.290E-02	−0.001	−0.069	−0.428	−0.417
84705	*GTPBP3*	GTP binding protein 3 (mitochondrial)	19p13.11	up	0.485	1.999E-02	−0.156	−0.048	−0.488	−1.061
84818	*IL17RC*	interleukin 17 receptor C	3p25.3	up	0.478	8.102E-03	−0.306	−0.009	−0.053	−0.194
10102	*TSFM*	Ts translation elongation factor, mitochondrial	12q14.1	up	0.475	4.873E-03	−0.170	−0.026	−0.951	−0.608
27	*ABL2*	c-abl oncogene 2, non-receptor tyrosine kinase	1q25.2	up	0.455	9.799E-03	−0.211	−0.281	−0.230	−0.102
55285	*RBM41*	RNA binding motif protein 41	Xq22.3	up	0.415	1.538E-02	−0.055	−0.215	−0.495	−0.559
57532	*NUFIP2*	nuclear fragile X mental retardation protein interacting protein 2	17q11.2	up	0.397	1.056E-02	−0.098	−0.256	−0.425	−0.904
84445	*LZTS2*	leucine zipper, putative tumor suppressor 2	10q24.31	up	0.394	4.155E-02	−0.174	−0.125	−0.288	−0.026
8243	*SMC1A*	structural maintenance of chromosomes 1A	Xp11.22	up	0.390	3.635E-02	−0.163	−0.061	−0.917	−0.297
54617	*INO80*	INO80 complex subunit	15q15.1	up	0.384	2.835E-03	−0.594	−0.006	−0.635	−0.350
7511	*XPNPEP1*	X-prolyl aminopeptidase (aminopeptidase P) 1, soluble	10q25.1	up	0.381	7.521E-03	−0.648	−0.272	−1.595	−1.701
23367	*LARP1*	La ribonucleoprotein domain family, member 1	5q33.2	up	0.377	4.155E-02	−0.049	−0.003	−0.091	−0.216
10146	*G3BP1*	GTPase activating protein (SH3 domain) binding protein 1	5q33.1	up	0.313	4.021E-02	−1.431	−0.040	−0.505	−0.475

### *UHRF1* was a direct target of *miR-145-5p* and *miR-145-3p* in BC cells

**Figure 4:**
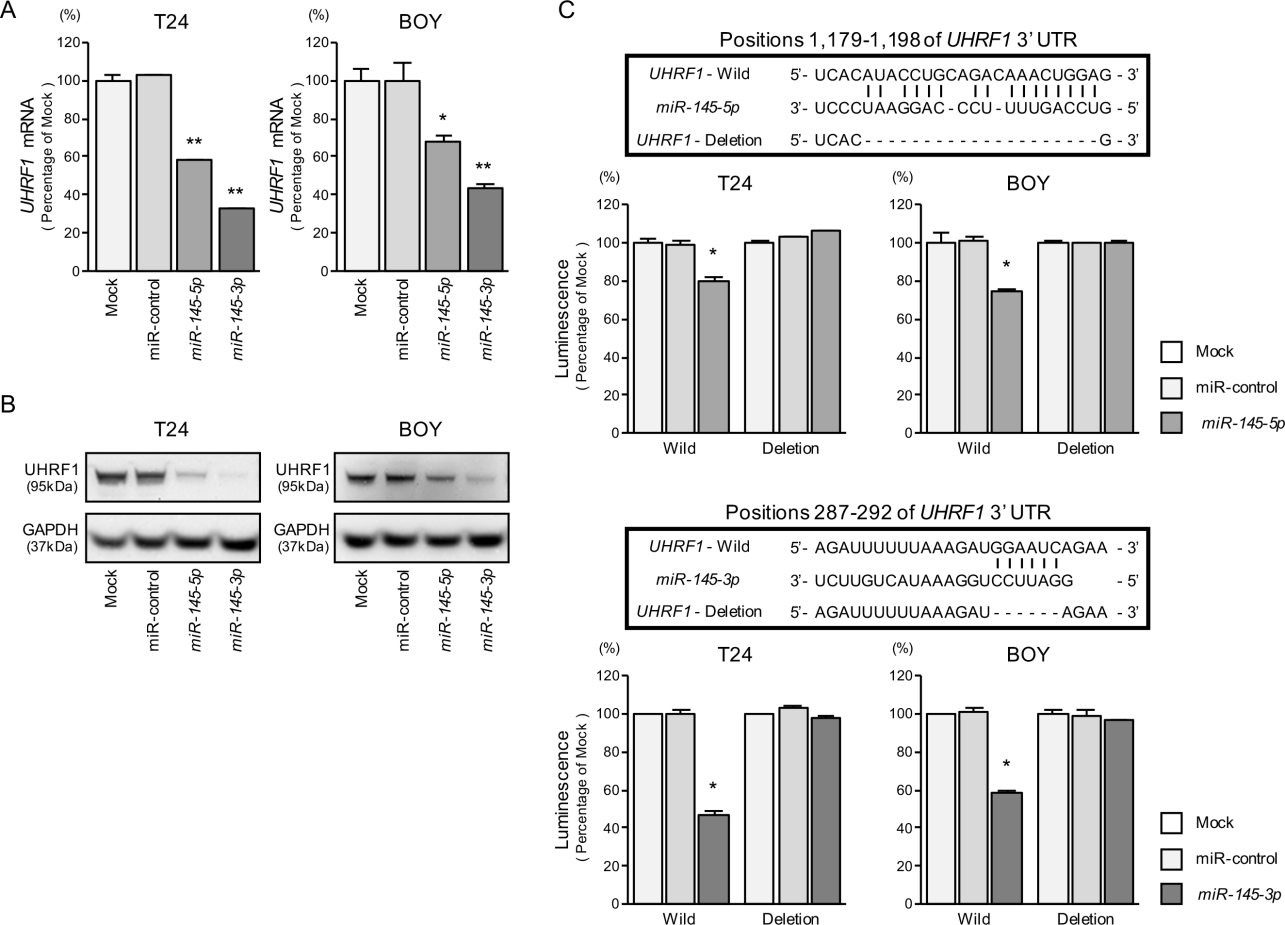
Direct regulation of UHRF1 by *miR-145-5p* and *miR-145-3p* (**A**) *UHRF1* mRNA expression was evaluated by qRT-PCR in T24 and BOY 72 hours after transfection with *miR-145-5p* and *miR-145-3p*. *GUSB* was used as an internal control. *P = 0.0036 and **P < 0.0001. (**B**) UHRF1 protein expression was evaluated by Western blot analyses in T24 and BOY 72–96 hours after transfection with *miR-145-5p* or *miR-145-3p*. GAPDH was used as a loading control. (**C**) *miR-145-5p* and *miR-145-3p* binding sites in the 3′ UTR of *UHRF1* mRNA. Dual Luciferase reporter assays using vectors encoding putative *miR-145-5p* and *miR-145-3p* target sites of the *UHRF* 3′ UTR (positions 1,179–1,198 and 287–292, respectively) for both wild-type and deleted regions. Normalized data were calculated as ratios of *Renilla*/firefly luciferase activities. *P < 0.0001.

We performed quantitative real-time RT-PCR (qRT-PCR) to validate that *miR-145-5p* and *miR-145-3p* repressed *UHRF1* mRNA expression in BC cell lines, and we did indeed observe that it was significantly reduced in transfectants of these miRNAs in comparison with mock or miR-control transfectants (*P* < 0.0001 and *P* = 0.0036, Figure 4A). The protein expression levels of UHRF1 were also repressed in the miRNAs transfectants (Figure 4B).

We carried out dual luciferase reporter assays in T24 and BOY cells to determine whether the *UHRF1* gene was directly regulated by *miR-145-5p*/*3p*. The microRNA.org database predicted that there was one binding site for *miR- 145-5p* in the 3′ UTR of *UHRF1* (position 1,179– 1,198); for *miR-145-3p*, there was a binding site in the 3′ UTR at position 287–292. We used vectors encoding the partial wild-type sequence of the 3′ UTR of the mRNA, including the predicted *miR-145-5p* or *miR-145*- *3p* target sites. We found that the luminescence intensity was significantly reduced by co-transfection with these miRNAs and the vector carrying the wild-type 3′ UTR, whereas no reduction of luminescence was observed by transfection with the deletion vector (binding site had been removed) (*P* < 0.0001, Figure 4C). These suggested that either of *miR-145-5p* and *miR-145-3p* were directly bounded to specific sites in the 3′ UTR of *UHRF1* mRNA.

### Effects of silencing *UHRF1* in BC cell lines

**Figure 5:**
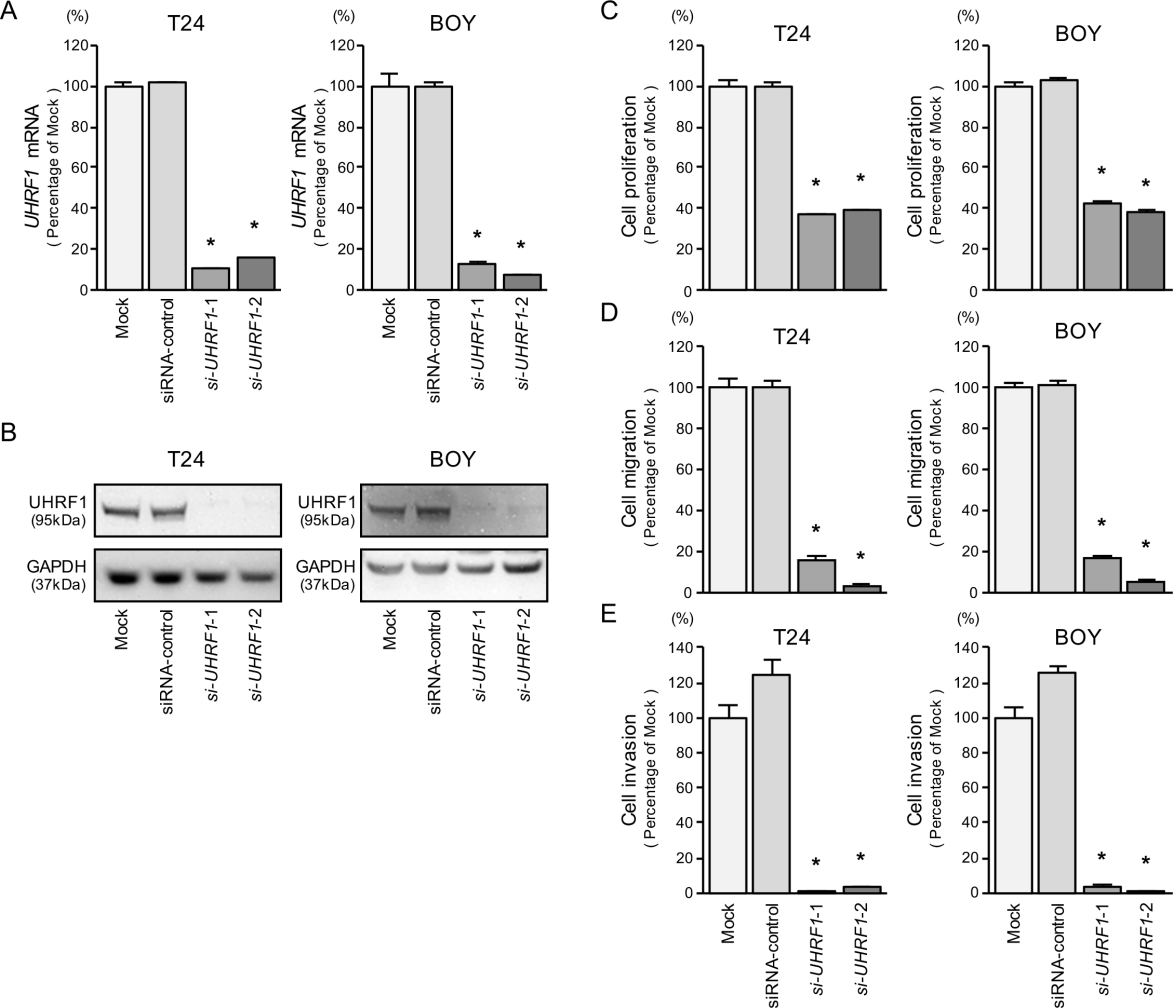
*UHRF1* mRNA and protein expression after *si-UHRF1* transfection and effects of UHRF1 silencing in BC cell lines (**A**) *UHRF1* mRNA expression was evaluated by qRT-PCR in T24 and BOY 72 hours after transfection with *si-UHRF1*-1 and *si-UHRF1*-2. *GUSB* was used as an internal control. (**B**) UHRF1 protein expression was evaluated by Western blot analysis in T24 and BOY 72 - 96 hours after transfection with *miR-145-5p* or *miR-145-3p*. GAPDH was used as a loading control. (**C**) Cell proliferation was determined with the XTT assays 72 hours after transfection with 10 nM *si-UHRF1*-1 or *si-UHRF1*-2. *P < 0.0001. (**D**) Cell migration activity was determined by wound-healing assays. *P < 0.0001. (**E**) Cell invasion activity was determined using Matrigel invasion assays. *P < 0.0001.

To investigate the functional role of *UHRF1* in BC cells, we carried out loss-of-function studies by using *si-UHRF1* transfectants. First, we evaluated the knockdown efficiency of *si-UHRF1* transfection in BC cell lines. In the present study, we used two types of *si- UHRF1* (*si- UHRF1*-1 and *si-UHRF1*-2). The qRT- PCR and Western blot analyses showed that both siRNAs effectively downregulated UHRF1 expression in both cell lines (Figure 5A and 5B).

XTT, cell migration, and invasion assays demonstrated that cell proliferation, cell migration, and cell invasion were inhibited in *si-UHRF1* transfectants in comparison with the mock or siRNA-control transfectant cells (each *P* < 0.0001, Figure 5C, 5D, and 5E).

**Figure 6:**
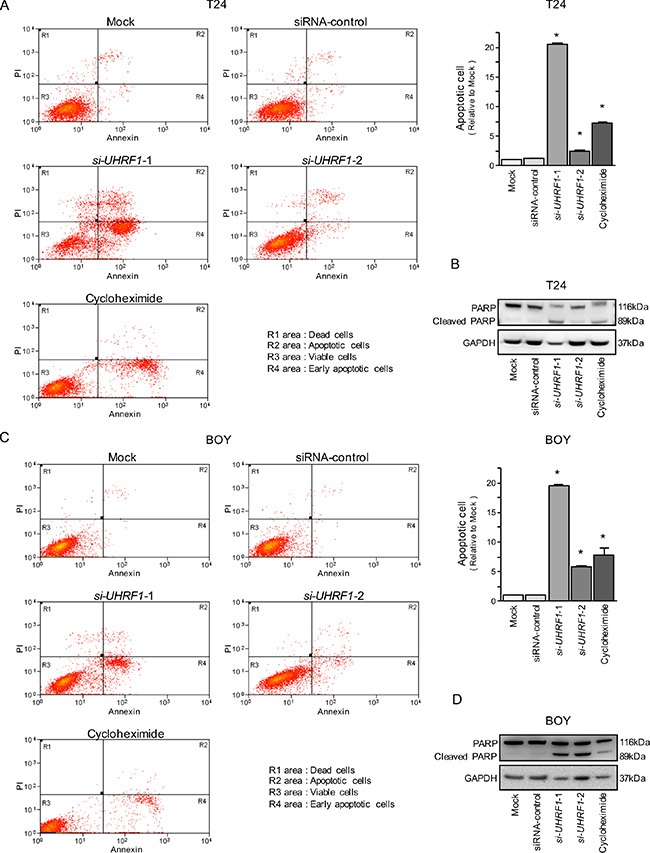
Effects of silencing *UHRF1* on apoptosis in BC cell lines (**A**, **C**) Apoptosis assays were carried out using flow cytometry. Early apoptotic cells are in area R4 and apoptotic cells are in area R2. The normalized ratios of the apoptotic cells are shown in the histogram. Cycloheximide (2 μg/mL) was used as a positive control. *P < 0.0001 (**B**, **D**) Western blot analyses for apoptotic markers (cleaved PARP) in BC cell lines. GAPDH was used as a loading control.

In the apoptosis assays, the apoptotic cell numbers were significantly greater in *si-UHRF1* transfectants than in mock or siRNA-control transfectants (Figure 6A and 6C). Western blot analyses showed that cleaved PARP expression was significantly increased in *si-UHRF1* transfectants compared with mock or siRNA-control transfectants (Figure 6B and 6D).

### Expression of *UHRF1* in BC clinical specimens

**Figure 7:**
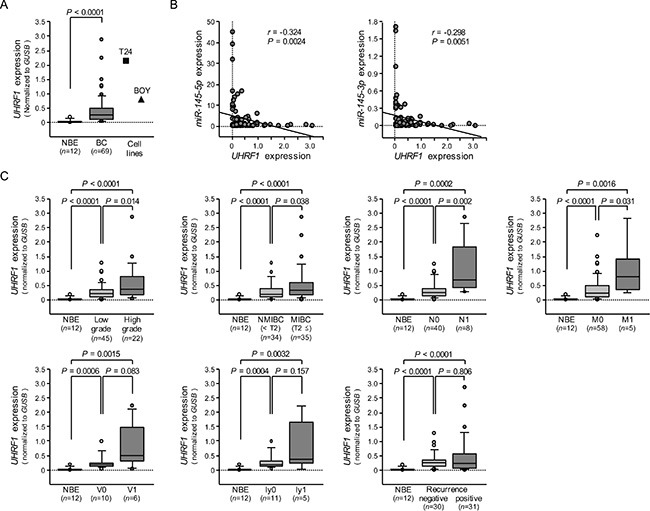
The expression level of *UHRF1* mRNA in BC clinical specimens and cell lines, and association of *UHRF1* expression with clinicopathological parameters (**A**) Expression levels of *UHRF1* in clinical specimens and BC cell lines were determined by qRT-PCR. Data were normalized to *GUSB* expression. (**B**) The correlated expression among *miR-145-5p*, *miR-145-3p*, and *UHRF1*. (**C**) Association of *UHRF1* expression with clinicopathological parameters. Relationships between two variables were analyzed using the Mann-Whitney *U* test.

**Figure 8:**
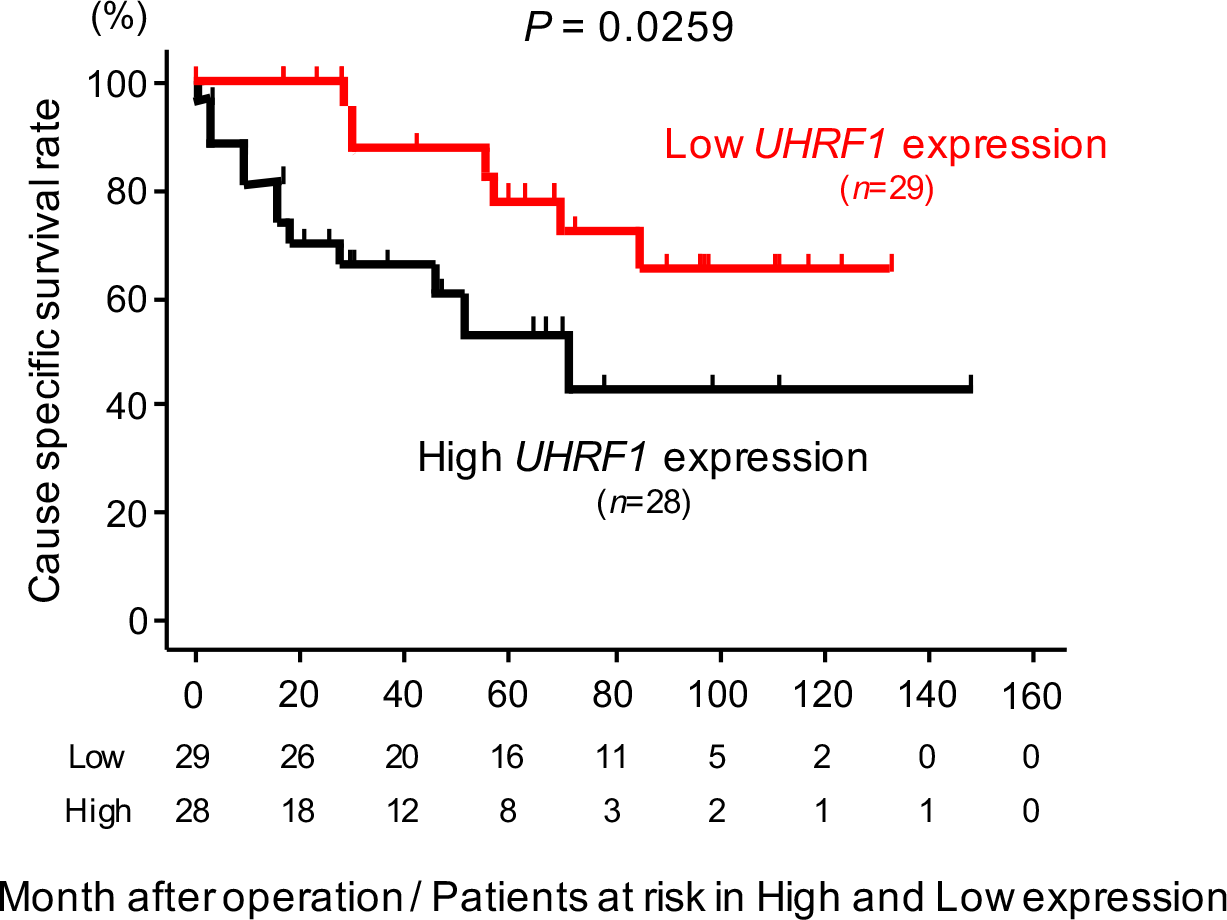
The association between the expression level of *UHRF1* and cause specific survival rate Kaplan-Meier survival curves for cause specific survival rates based on *UHRF1* expression in 57 BC patients. *P*-values were calculated using the log-rank test.

The qRT-PCR analyses showed that the expression level of *UHRF1* mRNA was significantly upregulated in 69 BC specimens and 2 BC cell lines compared with 12 NBE (*P* < 0.0001, Figure 7A). Spearman's rank test showed negative correlations between *miR-145-5p*/*miR-145*-*3p* expression and *UHRF1* mRNA expression (*r* = −0.324 and −0.298, *P* = 0.0024 and 0.0051, Figure 7B). As shown in Figure 7C, the expression level of *UHRF1* was significantly greater in high grade clinical BCs (*P* = 0.0135), MIBCs (T2 ≤) (*P* = 0.0379), BCs with positive lymph node invasion (N1) (*P* = 0.00182), and in BCs with positive distant metastasis (M1) (*P* = 0.0307) than in their counterparts. Kaplan-Meier analysis showed that the high *UHRF1* expression group had significantly lower cause specific survival probabilities compared to the low *UHRF1* expression group (*P* = 0.0259, Figure 8).

**Figure 9:**
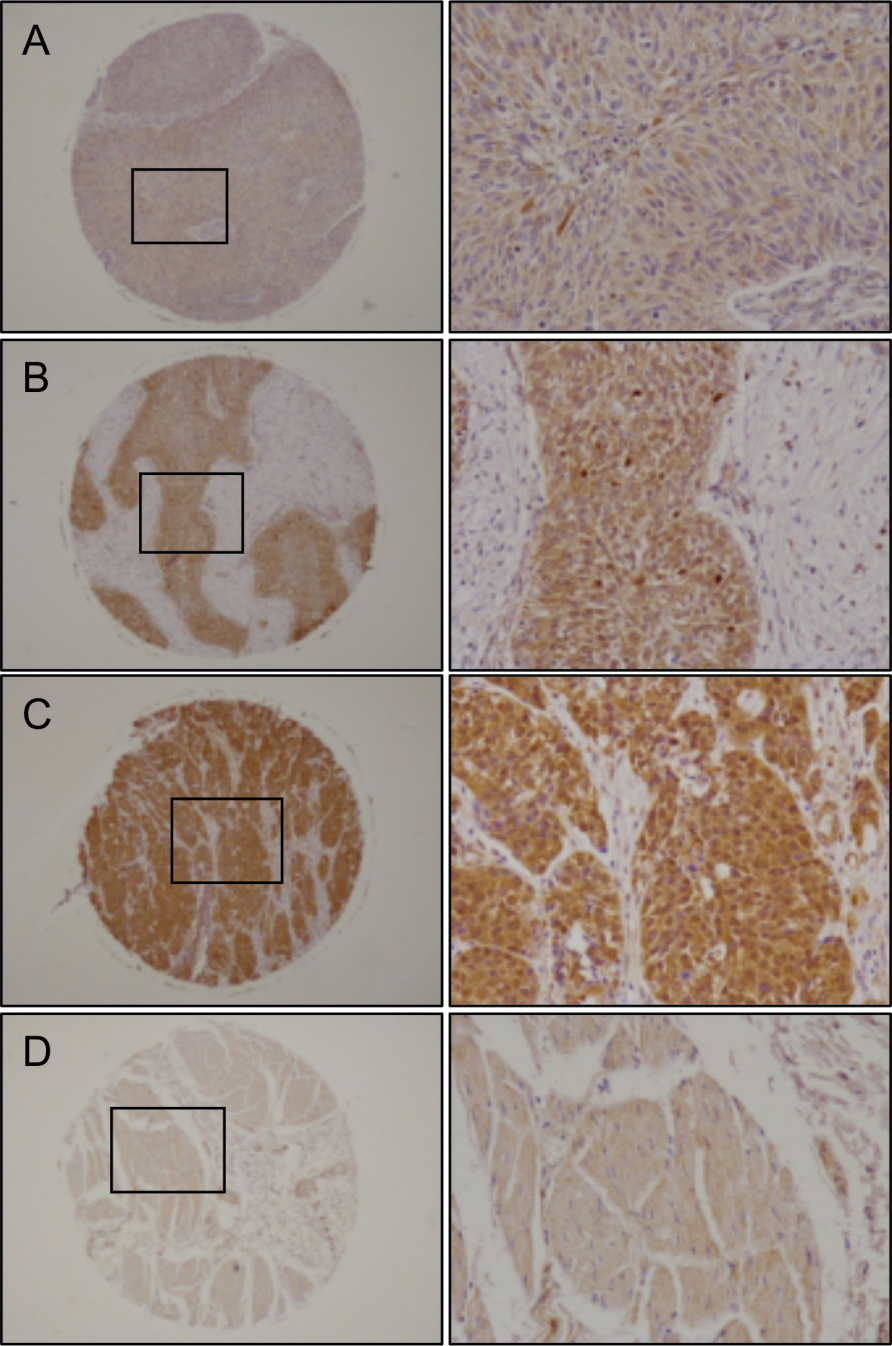
Immunohistochemical staining of UHRF1 in BC clinical specimens UHRF1 was expressed more strongly in several cancer lesions than in noncancerous tissues. Left panel, original magnification ×40; Right panel, original magnification ×200. (**A**) Positively stained tumor lesion (High grade, T2bN0M0), (**B**) Positively stained tumor lesion (High grade, T1N0M0), (**C**) Positively stained tumor lesion (Low grade, T3N0M0), (**D**) Negative staining in normal bladder tissue.

We validated the expression status of UHRF1 in BC clinical specimens using immunohistochemical staining. UHRF1 was expressed moderately or strongly in several cancer lesions, and normal bladder tissues stained weakly (Figure 9).

### Investigation of downstream genes regulated by *UHRF1* in BC cells

To identify the downstream genes regulated by *UHRF1*, genome-wide gene expression analyses and *in silico* analyses were performed in two BC cell lines transfected with *si-UHRF1*. A total of 533 genes were downregulated (log_2_ FC < −1.5) by *si-UHRF1* transfection, and a total of 704 genes were upregulated (log_2_ FC > 1.0) by *si-UHRF1* transfection compared with negative control cells (GEO, accession number: GSE77790). Among the downregulated genes in the *si-UHRF1* transfectants, 104 genes were upregulated in the BC clinical samples from GEO database (accession numbers: GSE11783, GSE31684), whereas among the upregulated genes, 62 genes were downregulated in the clinical BCs. These results imply that the 104 upregulated genes may act as oncogenes, and the 62 downregulated genes may act as tumor suppressors downstream from *UHRF1* in BC (Tables 2 and 3).

**Table 2:** Significantly downregulated genes by *si-UHRF1* in BC cell lines

Entrez Gene ID	Gene Symbol	Description	Genomic location	Gene Expression Omnibus (GSE11783 + GSE31684)			Expression in *si-UHRF1* transfectant (Log_2_ FC)	
				Expression	Log_2_FC	*P*-value	T24	BOY
7153	*TOP2A*	topoisomerase (DNA) II alpha 170kDa	17q21.2	up	6.312	1.049E-03	−1.880	−1.681
29128	*UHRF1*	ubiquitin-like with PHD and ring finger domains 1	19p13.3	up	4.984	1.049E-03	−3.213	−2.907
259266	*ASPM*	asp (abnormal spindle) homolog, microcephaly associated (Drosophila)	1q31.3	up	4.299	1.049E-03	−3.431	−3.444
332	*BIRC5*	baculoviral IAP repeat containing 5	17q25.3	up	4.110	1.049E-03	−2.258	−1.777
9928	*KIF14*	kinesin family member 14	1q32.1	up	3.866	1.049E-03	−3.294	−1.544
1063	*CENPF*	centromere protein F, 350/400kDa	1q41	up	3.576	1.049E-03	−2.613	−3.307
1894	*ECT2*	epithelial cell transforming 2	3q26.31	up	3.469	1.049E-03	−1.928	−1.813
55247	*NEIL3*	nei endonuclease VIII-like 3 (E. coli)	4q34.3	up	3.428	1.049E-03	−1.728	−2.065
9401	*RECQL4*	RecQ protein-like 4	8q24.3	up	3.414	1.049E-03	−1.751	−2.102
3832	*KIF11*	kinesin family member 11	10q23.33	up	3.356	1.049E-03	−2.299	−1.657
57082	*CASC5*	cancer susceptibility candidate 5	15q15.1	up	3.230	1.049E-03	−2.470	−2.188
151176	*FAM132B*	family with sequence similarity 132, member B	2q37.3	up	3.100	1.058E-03	−2.420	−2.184
151246	*SGOL2*	shugoshin-like 2 (S. pombe)	2q33.1	up	2.694	1.049E-03	−3.124	−2.407
1062	*CENPE*	centromere protein E, 312kDa	4q24	up	2.689	1.058E-03	−3.676	−3.218
23529	*CLCF1*	cardiotrophin-like cytokine factor 1	11q13.2	up	2.646	1.049E-03	−1.905	−2.363
81930	*KIF18A*	kinesin family member 18A	11p14.1	up	2.553	1.049E-03	−3.246	−2.128
7130	*TNFAIP6*	tumor necrosis factor, alpha-induced protein 6	2q23.3	up	2.531	2.835E-03	−1.795	−2.735
55502	*HES6*	hes family bHLH transcription factor 6	2q37.3	up	2.506	6.688E-03	−1.572	−1.508
5328	*PLAU*	plasminogen activator, urokinase	10q22.2	up	2.244	1.740E-03	−2.417	−1.791
9824	*ARHGAP11A*	Rho GTPase activating protein 11A	15q13.3	up	2.051	2.348E-03	−1.675	−1.613
23057	*NMNAT2*	nicotinamide nucleotide adenylyltransferase 2	1q25.3	up	2.050	1.247E-03	−1.707	−1.863
59285	*CACNG6*	calcium channel, voltage-dependent, gamma subunit 6	19q13.42	up	2.016	1.049E-03	−1.502	−1.763
675	*BRCA2*	breast cancer 2, early onset	13q13.1	up	2.015	1.049E-03	−1.764	−2.356
6524	*SLC5A2*	solute carrier family 5 (sodium/glucose cotransporter), member 2	16p11.2	up	1.900	1.214E-03	−1.855	−1.569
79412	*KREMEN2*	kringle containing transmembrane protein 2	16p13.3	up	1.893	1.348E-03	−2.309	−1.796
6274	*S100A3*	S100 calcium binding protein A3	1q21.3	up	1.825	8.102E-03	−2.215	−1.848
5331	*PLCB3*	phospholipase C, beta 3 (phosphatidylinositol-specific)	11q13.1	up	1.790	1.049E-03	−2.219	−1.735
55349	*CHDH*	choline dehydrogenase	3p21.1	up	1.743	1.049E-03	−1.926	−2.008
811	*CALR*	calreticulin	19p13.2	up	1.652	1.049E-03	−1.554	−1.500
4987	*OPRL1*	opiate receptor-like 1	20q13.33	up	1.627	2.626E-03	−1.927	−1.766
375248	*ANKRD36*	ankyrin repeat domain 36	2q11.2	up	1.530	8.102E-03	−3.873	−1.791
441054	*C4orf47*	chromosome 4 open reading frame 47	4q35.1	up	1.485	2.151E-02	−2.229	−2.522
201475	*RAB12*	RAB12, member RAS oncogene family	18p11.22	up	1.468	1.058E-03	−2.353	−2.947
286151	*FBXO43*	F-box protein 43	8q22.2	up	1.463	2.396E-02	−1.528	−2.082
9091	*PIGQ*	phosphatidylinositol glycan anchor biosynthesis, class Q	16p13.3	up	1.434	3.574E-03	−1.594	−1.693
81575	*APOLD1*	apolipoprotein L domain containing 1	12p13.1	up	1.354	1.808E-03	−2.237	−2.383
132320	*SCLT1*	sodium channel and clathrin linker 1	4q28.2	up	1.340	1.049E-03	−3.140	−3.098
100131211	*TMEM194B*	transmembrane protein 194B	2q32.2	up	1.325	1.049E-03	−1.573	−1.967
153642	*ARSK*	arylsulfatase family, member K	5q15	up	1.252	1.049E-03	−2.052	−1.875
21	*ABCA3*	ATP-binding cassette, sub-family A (ABC1), member 3	16p13.3	up	1.170	4.892E-02	−1.879	−1.831
55036	*CCDC40*	coiled-coil domain containing 40	17q25.3	up	1.160	1.049E-03	−1.562	−1.531
84259	*DCUN1D5*	DCN1, defective in cullin neddylation 1, domain containing 5	11q22.3	up	1.151	1.247E-03	−1.591	−1.993
80381	*CD276*	CD276 molecule	15q24.1	up	1.146	1.072E-03	−2.656	−2.096
6487	*ST3GAL3*	ST3 beta-galactoside alpha-2,3-sialyltransferase 3	1p34.1	up	1.139	1.049E-03	−1.828	−2.380
5351	*PLOD1*	procollagen-lysine, 2-oxoglutarate 5-dioxygenase 1	1p36.22	up	1.104	2.942E-03	−1.650	−1.570
343099	*CCDC18*	coiled-coil domain containing 18	1p22.1	up	1.075	1.578E-03	−3.521	−2.428
30818	*KCNIP3*	Kv channel interacting protein 3, calsenilin	2q11.1	up	1.069	2.723E-03	−3.678	−2.733
10051	*SMC4*	structural maintenance of chromosomes 4	3q25.33	up	1.066	1.578E-03	−2.612	−1.745
51427	*ZNF107*	zinc finger protein 107	7q11.21	up	1.040	1.316E-03	−2.527	−2.104
10592	*SMC2*	structural maintenance of chromosomes 2	9q31.1	up	1.032	6.688E-03	−3.520	−2.180
20	*ABCA2*	ATP-binding cassette, sub-family A (ABC1), member 2	9q34.3	up	0.965	1.372E-02	−1.511	−2.291
55183	*RIF1*	replication timing regulatory factor 1	2q23.3	up	0.960	1.058E-03	−1.712	−1.605
9898	*UBAP2L*	ubiquitin associated protein 2-like	1q21.3	up	0.952	1.049E-03	−1.587	−2.301
29780	*PARVB*	parvin, beta	22q13.31	up	0.952	1.096E-02	−3.288	−1.888
9585	*KIF20B*	kinesin family member 20B	10q23.31	up	0.933	5.720E-03	−2.282	−3.122
9534	*ZNF254*	zinc finger protein 254	19p12	up	0.920	3.863E-03	−2.072	−2.662
57520	*HECW2*	HECT, C2 and WW domain containing E3 ubiquitin protein ligase 2	2q32.3	up	0.884	3.179E-03	−1.838	−1.958
84083	*ZRANB3*	zinc finger, RAN-binding domain containing 3	2q21.3	up	0.873	1.578E-03	−1.987	−1.915
6498	*SKIL*	SKI-like proto-oncogene	3q26.2	up	0.859	1.808E-03	−2.709	−1.845
64770	*CCDC14*	coiled-coil domain containing 14	3q21.1	up	0.842	6.943E-03	−2.453	−1.711
254065	*BRWD3*	bromodomain and WD repeat domain containing 3	Xq21.1	up	0.808	1.393E-03	−1.852	−2.546
22973	*LAMB2P1*	laminin, beta 2 pseudogene 1	3p21.31	up	0.804	7.521E-03	−2.336	−2.311
7525	*YES1*	YES proto-oncogene 1, Src family tyrosine kinase	18p11.32	up	0.794	2.526E-03	−3.127	−2.099
1984	*EIF5A*	eukaryotic translation initiation factor 5A	17p13.1	up	0.793	5.486E-03	−2.297	−2.018
22852	*ANKRD26*	ankyrin repeat domain 26	10p12.1	up	0.787	3.303E-03	−2.798	−2.663
23322	*RPGRIP1L*	RPGRIP1-like	16q12.2	up	0.778	1.182E-02	−1.517	−1.806
79677	*SMC6*	structural maintenance of chromosomes 6	2p24.2	up	0.764	8.401E-03	−1.909	−2.083
84920	*ALG10*	ALG10, alpha-1,2-glucosyltransferase	12p11.1	up	0.763	6.688E-03	−1.828	−2.360
8570	*KHSRP*	KH-type splicing regulatory protein	19p13.3	up	0.762	3.303E-03	−1.767	−1.820
5819	*PVRL2*	poliovirus receptor-related 2 (herpesvirus entry mediator B)	19q13.32	up	0.757	9.078E-03	−3.014	−2.465
51575	*ESF1*	ESF1, nucleolar pre-rRNA processing protein, homolog (S. cerevisiae)	20p12.1	up	0.755	9.430E-03	−1.786	−1.732
51361	*HOOK1*	hook microtubule-tethering protein 1	1p32.1	up	0.689	3.067E-02	−2.156	−2.000
10198	*MPHOSPH9*	M-phase phosphoprotein 9	12q24.31	up	0.667	1.947E-03	−2.113	−1.502
4983	*OPHN1*	oligophrenin 1	Xq12	up	0.632	5.277E-03	−2.278	−1.747
4976	*OPA1*	optic atrophy 1 (autosomal dominant)	3q29	up	0.619	2.169E-03	−2.190	−1.526
168850	*ZNF800*	zinc finger protein 800	7q31.33	up	0.611	1.227E-02	−1.807	−1.867
26272	*FBXO4*	F-box protein 4	5p13.1	up	0.611	3.512E-02	−2.224	−2.445
7390	*UROS*	uroporphyrinogen III synthase	10q26.13	up	0.605	6.433E-03	−3.120	−2.062
4683	*NBN*	nibrin	8q21.3	up	0.590	5.720E-03	−2.986	−1.966
79670	*ZCCHC6*	zinc finger, CCHC domain containing 6	9q21.33	up	0.587	5.486E-03	−2.353	−1.839
79573	*TTC13*	tetratricopeptide repeat domain 13	1q42.2	up	0.587	6.943E-03	−1.740	−2.064
50840	*TAS2R14*	taste receptor, type 2, member 14	12p13.2	up	0.574	1.598E-02	−1.947	−1.509
79042	*TSEN34*	TSEN34 tRNA splicing endonuclease subunit	19q13.42	up	0.570	1.138E-02	−2.455	−1.761
6801	*STRN*	striatin, calmodulin binding protein	2p22.2	up	0.563	2.723E-03	−1.964	−2.434
3597	*IL13RA1*	interleukin 13 receptor, alpha 1	Xq24	up	0.552	2.075E-02	−2.460	−2.403
147657	*ZNF480*	zinc finger protein 480	19q13.41	up	0.547	3.893E-02	−3.434	−3.276
8683	*SRSF9*	serine/arginine-rich splicing factor 9	12q24.31	up	0.534	1.227E-02	−1.523	−2.098
252983	*STXBP4*	syntaxin binding protein 4	17q22	up	0.516	2.151E-02	−1.776	−1.599
284325	*C19orf54*	chromosome 19 open reading frame 54	19q13.2	up	0.510	4.734E-02	−1.614	−2.171
91147	*TMEM67*	transmembrane protein 67	8q22.1	up	0.509	9.799E-03	−1.647	−2.069
114799	*ESCO1*	establishment of sister chromatid cohesion N-acetyltransferase 1	18q11.2	up	0.495	4.873E-03	−2.173	−2.401
57670	*KIAA1549*	KIAA1549	7q34	up	0.480	4.582E-02	−2.127	−1.789
6103	*RPGR*	retinitis pigmentosa GTPase regulator	Xp11.4	up	0.467	3.290E-02	−1.583	−2.025
5700	*PSMC1*	proteasome (prosome, macropain) 26S subunit, ATPase, 1	14q32.11	up	0.449	1.274E-02	−1.639	−1.711
253260	*RICTOR*	RPTOR independent companion of MTOR, complex 2	5p13.1	up	0.442	2.666E-02	−2.458	−1.683
23241	*PACS2*	phosphofurin acidic cluster sorting protein 2	14q32.33	up	0.442	3.179E-03	−3.416	−2.028
27154	*BRPF3*	bromodomain and PHD finger containing, 3	6p21.31	up	0.440	5.720E-03	−1.772	−2.598
7703	*PCGF2*	polycomb group ring finger 2	17q12	up	0.439	2.865E-02	−1.828	−1.974
51105	*PHF20L1*	PHD finger protein 20-like 1	8q24.22	up	0.383	9.078E-03	−3.492	−2.007
57697	*FANCM*	Fanconi anemia, complementation group M	14q21.2	up	0.364	3.067E-02	−1.648	−1.627
9730	*VPRBP*	Vpr (HIV-1) binding protein	3p21.2	up	0.363	2.075E-02	−2.342	−1.568
5378	*PMS1*	PMS1 postmeiotic segregation increased 1 (S. cerevisiae)	2q32.2	up	0.350	4.734E-02	−2.701	−1.616
255520	*ELMOD2*	ELMO/CED-12 domain containing 2	4q31.1	up	0.334	4.582E-02	−2.360	−1.637
80124	*VCPIP1*	valosin containing protein (p97)/p47 complex interacting protein 1	8q13.1	up	0.304	3.893E-02	−3.107	−2.286

**Table 3:** Significantly upregulated genes by *si-UHRF1* in BC cell lines

Entrez Gene ID	Gene Symbol	Description	Genomic location	Gene Expression Omnibus (GSE11783 + GSE31684)			Expression in *si-UHRF1* transfectant (Log_2_ FC)	
				Expression	Log_2_FC	*P*-value	T24	BOY
3043	*HBB*	hemoglobin, beta	11p15.4	down	−3.263	1.214E-03	1.204	2.109
137835	*TMEM71*	transmembrane protein 71	8q24.22	down	−2.428	4.873E-03	2.813	3.920
8639	*AOC3*	amine oxidase, copper containing 3	17q21.31	down	−2.188	1.434E-03	1.907	3.140
1408	*CRY2*	cryptochrome circadian clock 2	11p11.2	down	−2.141	1.058E-03	2.134	2.108
7644	*ZNF91*	zinc finger protein 91	19p12	down	−2.058	1.155E-03	1.435	2.063
197257	*LDHD*	lactate dehydrogenase D	16q23.1	down	−1.626	2.965E-02	1.844	1.362
316	*AOX1*	aldehyde oxidase 1	2q33.1	down	−1.601	2.169E-03	1.841	1.049
26051	*PPP1R16B*	protein phosphatase 1, regulatory subunit 16B	20q11.23	down	−1.547	6.688E-03	1.076	1.198
63976	*PRDM16*	PR domain containing 16	1p36.32	down	−1.439	2.075E-02	2.639	3.846
254827	*NAALADL2*	N-acetylated alpha-linked acidic dipeptidase-like 2	3q26.31	down	−1.313	4.873E-03	1.621	3.168
154	*ADRB2*	adrenoceptor beta 2, surface	5q32	down	−1.242	9.799E-03	2.384	2.302
10477	*UBE2E3*	ubiquitin-conjugating enzyme E2E 3	2q31.3	down	−1.117	1.135E-03	1.053	2.755
7099	*TLR4*	toll-like receptor 4	9q33.1	down	−1.053	6.943E-03	1.402	2.356
57478	*USP31*	ubiquitin specific peptidase 31	16p12.2	down	−1.037	4.169E-03	1.570	1.234
57185	*NIPAL3*	NIPA-like domain containing 3	1p36.11	down	−0.986	1.316E-03	1.329	1.189
30815	*ST6GALNAC6*	ST6 (alpha-N-acetyl-neuraminyl-2,3-beta-galactosyl-1,3)-N-acetylgalactosaminide alpha-2,6-sialyltransferase 6	9q34.11	down	−0.936	1.660E-02	1.093	2.348
29915	*HCFC2*	host cell factor C2	12q23.3	down	−0.928	1.393E-03	1.304	1.296
54741	*LEPROT*	leptin receptor overlapping transcript	1p31.3	down	−0.893	1.049E-03	1.280	2.248
7779	*SLC30A1*	solute carrier family 30 (zinc transporter), member 1	1q32.3	down	−0.879	8.736E-03	1.267	1.262
79027	*ZNF655*	zinc finger protein 655	7q22.1	down	−0.863	1.393E-03	1.570	1.589
64344	*HIF3A*	hypoxia inducible factor 3, alpha subunit	19q13.32	down	−0.845	1.016E-02	1.284	2.411
79844	*ZDHHC11*	zinc finger, DHHC-type containing 11	5p15.33	down	−0.834	3.176E-02	1.505	1.890
79815	*NIPAL2*	NIPA-like domain containing 2	8q22.2	down	−0.825	6.688E-03	1.929	1.259
7923	*HSD17B8*	hydroxysteroid (17-beta) dehydrogenase 8	6p21.32	down	−0.821	3.512E-02	2.657	3.759
8629	*JRK*	Jrk homolog (mouse)	8q24.3	down	−0.820	1.740E-03	1.358	2.076
79591	*C10orf76*	chromosome 10 open reading frame 76	10q24.32	down	−0.812	1.808E-03	1.099	1.917
599	*BCL2L2*	BCL2-like 2	14q11.2	down	−0.775	2.835E-03	1.384	1.730
412	*STS*	steroid sulfatase (microsomal), isozyme S	Xp22.31	down	−0.770	1.372E-02	1.440	1.471
56900	*TMEM167B*	transmembrane protein 167B	1p13.3	down	−0.755	2.626E-03	2.282	2.366
23509	*POFUT1*	protein O-fucosyltransferase 1	20q11.21	down	−0.747	1.274E-02	1.400	2.132
25923	*ATL3*	atlastin GTPase 3	11q12.3	down	−0.727	3.290E-02	1.179	1.907
79669	*C3orf52*	chromosome 3 open reading frame 52	3q13.2	down	−0.708	4.021E-02	1.200	1.482
55844	*PPP2R2D*	protein phosphatase 2, regulatory subunit B, delta	10q26.3	down	−0.691	2.666E-02	1.422	1.303
5939	*RBMS2*	RNA binding motif, single stranded interacting protein 2	12q13.3	down	−0.626	5.943E-03	1.193	1.438
6158	*RPL28*	ribosomal protein L28	19q13.42	down	−0.618	1.808E-03	2.026	3.427
2145	*EZH1*	enhancer of zeste 1 polycomb repressive complex 2 subunit	17q21.2	down	−0.618	1.393E-03	1.391	1.171
388969	*C2orf68*	chromosome 2 open reading frame 68	2p11.2	down	−0.611	3.435E-03	1.309	1.192
55422	*ZNF331*	zinc finger protein 331	19q13.42	down	−0.594	1.725E-02	2.855	2.230
92400	*RBM18*	RNA binding motif protein 18	9q33.2	down	−0.594	8.401E-03	1.172	2.001
80017	*C14orf159*	chromosome 14 open reading frame 159	14q32.11	down	−0.590	1.182E-02	1.072	1.748
7556	*ZNF10*	zinc finger protein 10	12q24.33	down	−0.563	1.480E-02	1.592	1.127
55957	*LIN37*	lin-37 DREAM MuvB core complex component	19q13.12	down	−0.543	1.857E-02	1.002	1.205
84267	*C9orf64*	chromosome 9 open reading frame 64	9q21.32	down	−0.543	5.720E-03	1.215	1.299
8799	*PEX11B*	peroxisomal biogenesis factor 11 beta	1q21.1	down	−0.535	4.679E-03	1.083	1.163
8790	*FPGT*	fucose-1-phosphate guanylyltransferase	1p31.1	down	−0.524	2.075E-02	1.680	1.222
6992	*PPP1R11*	protein phosphatase 1, regulatory (inhibitor) subunit 11	6p22.1	down	−0.517	6.433E-03	1.104	1.329
116224	*FAM122A*	family with sequence similarity 122A	9q21.11	down	−0.507	2.169E-03	1.231	1.549
51710	*ZNF44*	zinc finger protein 44	19p13.2	down	−0.499	1.372E-02	2.385	1.001
7265	*TTC1*	tetratricopeptide repeat domain 1	5q33.3	down	−0.487	1.182E-02	1.109	1.112
80213	*TM2D3*	TM2 domain containing 3	15q26.3	down	−0.485	1.182E-02	1.342	1.742
81631	*MAP1LC3B*	microtubule-associated protein 1 light chain 3 beta	16q24.2	down	−0.480	1.725E-02	1.210	2.109
6016	*RIT1*	Ras-like without CAAX 1	1q22	down	−0.473	2.666E-02	1.556	1.432
7247	*TSN*	translin	2q14.3	down	−0.467	4.582E-02	1.101	1.496
167227	*DCP2*	decapping mRNA 2	5q22.2	down	−0.447	1.016E-02	1.284	1.104
11046	*SLC35D2*	solute carrier family 35 (UDP-GlcNAc/UDP-glucose transporter), member D2	9q22.32	down	−0.431	1.227E-02	1.318	1.340
54946	*SLC41A3*	solute carrier family 41, member 3	3q21.2	down	−0.402	4.294E-02	1.526	1.988
7799	*PRDM2*	PR domain containing 2, with ZNF domain	1p36.21	down	−0.384	7.805E-03	1.438	1.294
6651	*SON*	SON DNA binding protein	21q22.11	down	−0.374	5.486E-03	1.126	1.155
80255	*SLC35F5*	solute carrier family 35, member F5	2q14.1	down	−0.369	4.441E-02	1.143	1.619
55197	*RPRD1A*	regulation of nuclear pre-mRNA domain containing 1A	18q12.2	down	−0.364	3.893E-02	1.480	1.761
91603	*ZNF830*	zinc finger protein 830	17q12	down	−0.358	2.075E-02	1.040	1.085
5094	*PCBP2*	poly(rC) binding protein 2	12q13.13	down	−0.286	4.734E-02	1.454	1.158

**Figure 10:**
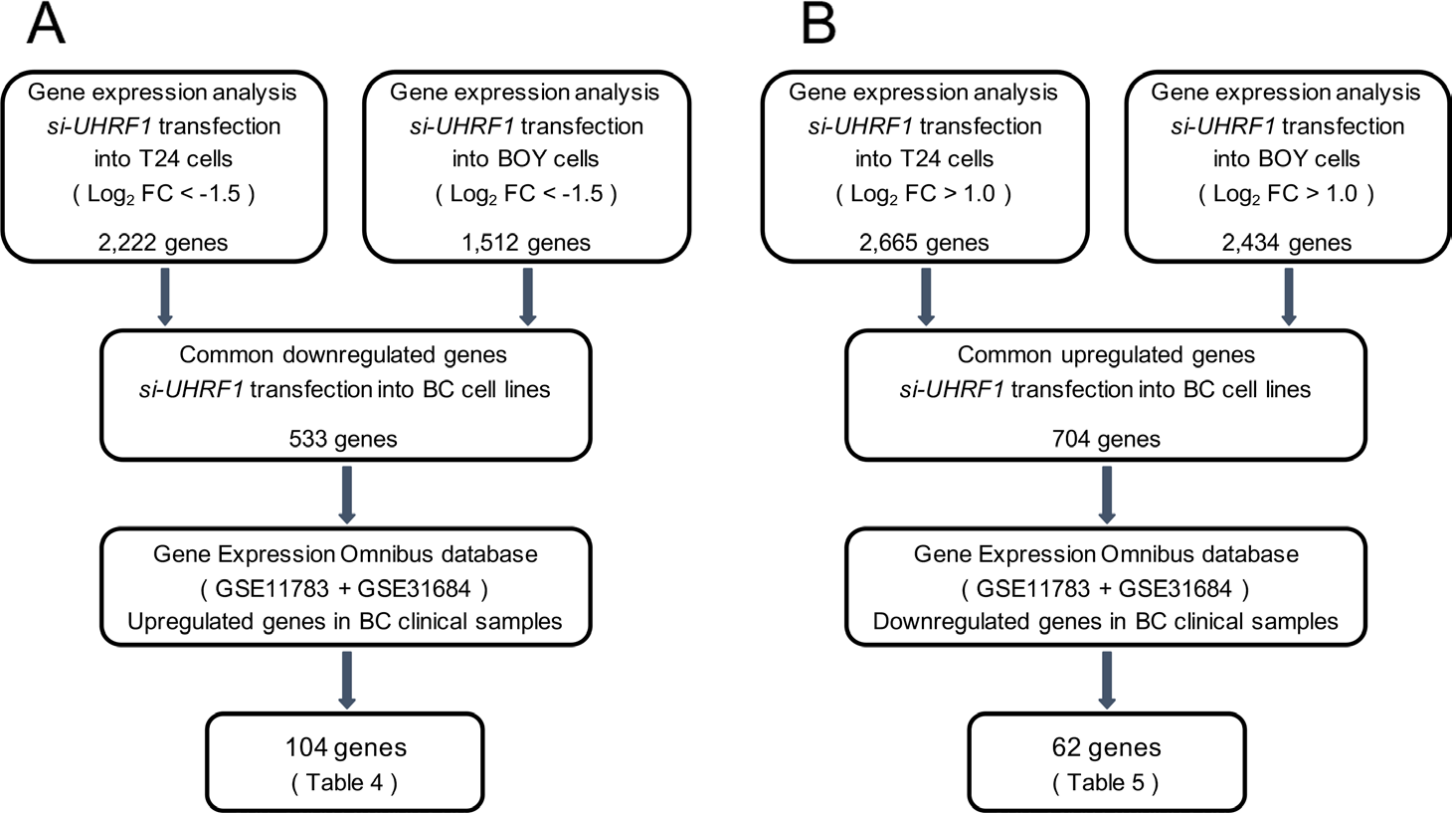
Flow chart demonstrating the strategy for analysis of genes regulated by *UHRF1* (**A**) A total of 2,222 and 1,512 downregulated genes in expression analyses of *si-UHRF1* transfectants of BC cell lines (T24 and BOY, respectively) were selected. We then analyzed 533 common downregulated genes by using available GEO data sets (GSE11783 + GSE31684). The analyses showed that 104 genes were significantly upregulated in BC specimens compared with NBE. (**B**) A total of 2,665 and 2,434 upregulated genes in expression analysis of *si-UHRF1* transfectants of BC cell lines (T24 and BOY, respectively) were selected. We then analyzed 704 common upregulated genes by using GEO data sets. The analyses showed that 62 genes were significantly downregulated in BC specimens compared with NBE.

To further investigate the *UHRF1* downstream genes, we performed the classification of these candidate genes to known molecular pathways by using DAVID program (https://david.ncifcrf.gov/). Classification strategy of downstream genes by *si-UHRF1* transfectants is shown in Figure 10A and 10B. Significantly upregulated and downregulated pathways and their involved genes are indicated in Tables 4 and 5. Several genes were classified into biological process categories and a variety of biological pathways, “M phase”, “cell cycle”, and “cell cycle phase” were significantly downregulated by *si- UHRF1* transfectants (Table 4).

**Table 4:** Downregulated genes by *si-UHRF1* were classified by DAVID program

Biological process	Number of genes	*P*-Value	Genes
M phase	15	8.10E-09	*ASPM, BIRC5, BRCA2, CENPE, CENPF, FBXO43, KIF11, KIF18A, KIF20B, MPHOSPH9, NBN, SGOL2, SMC2, SMC4, VCPIP1*
cell cycle	20	1.10E-07	*ASPM, BIRC5, BRCA2, CALR, CENPE, CENPF, ESCO1, FBXO43, KIF11, KIF18A, KIF20B, MPHOSPH9, NBN, PSMC1, RIF1, SGOL2, SMC2, SMC4, UHRF1, VCPIP1*
cell cycle phase	15	1.40E-07	*ASPM, BIRC5, BRCA2, CENPE, CENPF, FBXO43, KIF11, KIF18A, KIF20B, MPHOSPH9, NBN, SGOL2, SMC2, SMC4, VCPIP1*
cell cycle process	17	1.90E-07	*ASPM, BIRC5, BRCA2, CALR, CENPE, CENPF, FBXO43, KIF11, KIF18A, KIF20B, MPHOSPH9, NBN, PSMC1, SGOL2, SMC2, SMC4, VCPIP1*
chromosome segregation	8	5.20E-07	*BIRC5, CENPE, CENPF, KIF18A, SGOL2, SMC2, SMC4, TOP2A*
M phase of mitotic cell cycle	11	8.50E-07	*ASPM, BIRC5, CENPE, CENPF, KIF11, KIF18A, KIF20B, MPHOSPH9, SMC2, SMC4, VCPIP1*
organelle fission	11	1.00E-06	*ASPM, BIRC5, CENPE, CENPF, KIF11, KIF18A, KIF20B, OPA1, SMC2, SMC4, VCPIP1*
mitosis	10	6.40E-06	*ASPM, BIRC5, CENPE, CENPF, KIF11, KIF18A, KIF20B, SMC2, SMC4, VCPIP1*
nuclear division	10	6.40E-06	*ASPM, BIRC5, CENPE, CENPF, KIF11, KIF18A, KIF20B, SMC2, SMC4, VCPIP1*
mitotic cell cycle	12	1.20E-05	*ASPM, BIRC5, CENPE, CENPF, KIF11, KIF18A, KIF20B, MPHOSPH9, PSMC1, SMC2, SMC4, VCPIP1*
DNA repair	10	4.90E-05	*BRCA2, ESCO1, FANCM, NBN, NEIL3, PMS1, RECQL4, SMC6, TOP2A, UHRF1*
cell division	10	6.50E-05	*ASPM, BIRC5, BRCA2, CENPE, CENPF, KIF11, KIF20B, SGOL2, SMC2, SMC4*
response to DNA damage stimulus	11	7.40E-05	*BRCA2, ESCO1, FANCM, NBN, NEIL3, PMS1, RECQL4, RIF1, SMC6, TOP2A, UHRF1*
establishment of chromosome localization	4	8.90E-05	*BIRC5, CENPE, CENPF, KIF18A*
chromosome localization	4	8.90E-05	*BIRC5, CENPE, CENPF, KIF18A*
chromosome organization	12	1.40E-04	*BRCA2, BRPF3, CENPE, CENPF, FBXO4, KIF18A, NBN, PCGF2, SGOL2, SMC2, SMC4, TOP2A*
DNA metabolic process	12	2.00E-04	*BRCA2, CENPF, ESCO1, FANCM, FBXO4, NBN, NEIL3, PMS1, RECQL4, SMC6, TOP2A, UHRF1*
microtubule-based movement	6	5.80E-04	*CENPE, KIF11, KIF14, KIF18A, KIF20B, OPA1*
regulation of cell cycle process	6	6.00E-04	*BIRC5, BRCA2, CALR, CENPE, CENPF, KIF20B*
microtubule-based process	8	7.90E-04	*BRCA2, CENPE, HOOK1, KIF11, KIF14, KIF18A, KIF20B, OPA1*
mitotic sister chromatid segregation	4	1.30E-03	*CENPE, KIF18A, SMC2, SMC4*
sister chromatid segregation	4	1.40E-03	*CENPE, KIF18A, SMC2, SMC4*
metaphase plate congression	3	1.90E-03	*CENPE, CENPF, KIF18A*
cellular response to stress	11	2.00E-03	*BRCA2, ESCO1, FANCM, NBN, NEIL3, PMS1, RECQL4, RIF1, SMC6, TOP2A, UHRF1*
regulation of mitotic cell cycle	6	2.20E-03	*BIRC5, BRCA2, CENPE, CENPF, KIF20B, NBN*
organelle localization	5	2.20E-03	*ASPM, BIRC5, CENPE, CENPF, KIF18A*
spindle checkpoint	3	2.20E-03	*BIRC5, CENPE, CENPF*
positive regulation of cell cycle	4	4.80E-03	*BIRC5, BRCA2, CALR, CENPE*
establishment of organelle localization	4	8.20E-03	*BIRC5, CENPE, CENPF, KIF18A*
chromosome condensation	3	9.70E-03	*SMC2, SMC4, TOP2A*
glucose transport	3	1.30E-02	*SLC5A2, STXBP4, YES1*
hexose transport	3	1.40E-02	*SLC5A2, STXBP4, YES1*
regulation of cell cycle	7	1.40E-02	*BIRC5, BRCA2, CALR, CENPE, CENPF, KIF20B, NBN*
monosaccharide transport	3	1.50E-02	*SLC5A2, STXBP4, YES1*
negative regulation of neuron differentiation	3	1.70E-02	*ASPM, CALR, NBN*
cell cycle checkpoint	4	1.70E-02	*BIRC5, CENPE, CENPF, NBN*
kinetochore assembly	2	1.80E-02	*CENPE, CENPF*
meiosis	4	2.10E-02	*BRCA2, FBXO43, NBN, SGOL2*
M phase of meiotic cell cycle	4	2.10E-02	*BRCA2, FBXO43, NBN, SGOL2*
meiotic cell cycle	4	2.20E-02	*BRCA2, FBXO43, NBN, SGOL2*
germ cell development	4	2.30E-02	*BRCA2, CASC5, HOOK1, PVRL2*
kinetochore organization	2	2.40E-02	*CENPE, CENPF*
DNA recombination	4	2.50E-02	*BRCA2, NBN, RECQL4, SMC6*
mitotic cell cycle checkpoint	3	2.70E-02	*CENPE, CENPF, NBN*
centromere complex assembly	2	3.50E-02	*CENPE, CENPF*
spermatid development	3	4.10E-02	*CASC5, HOOK1, PVRL2*
regulation of nuclear division	3	4.40E-02	*CENPE, CENPF, KIF20B*
regulation of mitosis	3	4.40E-02	*CENPE, CENPF, KIF20B*
negative regulation of macromolecule biosynthetic process	8	4.50E-02	*BRCA2, CALR, CD276, CENPF, KCNIP3, PCGF2, SKIL, ZNF254*
spermatid differentiation	3	4.60E-02	*CASC5, HOOK1, PVRL2*
cytoskeleton organization	7	4.60E-02	*BRCA2, CALR, HOOK1, KIF11, KIF18A, OPHN1, RICTOR*
negative regulation of cellular biosynthetic process	8	5.10E-02	*BRCA2, CALR, CD276, CENPF, KCNIP3, PCGF2, SKIL, ZNF254*
positive regulation of cellular protein metabolic process	5	5.10E-02	*CLCF1, EIF5A, FBXO4, PSMC1, RICTOR*
carbohydrate transport	3	5.20E-02	*SLC5A2, STXBP4, YES1*
mitotic metaphase plate congression	2	5.30E-02	*CENPE, KIF18A*
regulation of DNA replication	3	5.30E-02	*BRCA2, CALR, NBN*
double-strand break repair	3	5.30E-02	*BRCA2, NBN, RECQL4*
negative regulation of biosynthetic process	8	5.50E-02	*BRCA2, CALR, CD276, CENPF, KCNIP3, PCGF2, SKIL, ZNF254*
positive regulation of protein metabolic process	5	5.80E-02	*CLCF1, EIF5A, FBXO4, PSMC1, RICTOR*
microtubule cytoskeleton organization	4	5.80E-02	*BRCA2, HOOK1, KIF11, KIF18A*
negative regulation of mitotic metaphase/anaphase transition	2	6.40E-02	*CENPE, CENPF*
blastocyst growth	2	6.40E-02	*BRCA2, NBN*
mitotic cell cycle spindle assembly checkpoint	2	6.40E-02	*CENPE, CENPF*
positive regulation of mitotic cell cycle	2	7.00E-02	*BIRC5, BRCA2*
negative regulation of mitosis	2	7.00E-02	*CENPE, CENPF*
negative regulation of nuclear division	2	7.00E-02	*CENPE, CENPF*
negative regulation of macromolecule metabolic process	9	7.20E-02	*BRCA2, CALR, CD276, CENPF, KCNIP3, PCGF2, PSMC1, SKIL, ZNF254*
reproductive cellular process	4	7.30E-02	*BRCA2, CASC5, HOOK1, PVRL2*
mitotic chromosome condensation	2	7.50E-02	*SMC2, SMC4*
negative regulation of transcription from RNA polymerase II promoter	5	7.50E-02	*CALR, KCNIP3, PCGF2, SKIL, ZNF254*
protein localization	10	8.00E-02	*CALR, CENPE, CENPF, EIF5A, HOOK1, KIF18A, RAB12, RPGR, SGOL2, STXBP4*
negative regulation of nucleobase, nucleoside, nucleotide and nucleic acid metabolic process	7	8.60E-02	*BRCA2, CALR, CENPF, KCNIP3, PCGF2, SKIL, ZNF254*
establishment of protein localization	9	8.90E-02	*CALR, CENPE, CENPF, EIF5A, HOOK1, KIF18A, RAB12, RPGR, STXBP4*
in utero embryonic development	4	8.90E-02	*BRCA2, NBN, PCGF2, RPGRIP1L*
negative regulation of nitrogen compound metabolic process	7	9.10E-02	*BRCA2, CALR, CENPF, KCNIP3, PCGF2, SKIL, ZNF254*
positive regulation of cellular component organization	4	9.50E-02	*CALR, CENPE, EIF5A, RICTOR*
developmental growth	3	9.60E-02	*BRCA2, NBN, PLAU*

**Table 5:** Upregulated genes by *si-UHRF1* were classified by DAVID program

Biological process	Number of genes	*P*-Value	Genes
regulation of transcription	15	1.40E-02	*CRY2, ADRB2, EZH1, HCFC2, HIF3A, JRK, POFUT1, PRDM16, PRDM2, TLR4, ZNF10, ZNF331, ZNF44, ZNF655, ZNF91*
regulation of transcription, DNA-dependent	10	7.00E-02	*ADRB2, HCFC2, HIF3A, PRDM16, PRDM2, ZNF10, ZNF331, ZNF44, ZNF655, ZNF91*
regulation of RNA metabolic process	10	7.90E-02	*ADRB2, HCFC2, HIF3A, PRDM16, PRDM2, ZNF10, ZNF331, ZNF44, ZNF655, ZNF91*
negative regulation of myeloid leukocyte differentiation	2	4.90E-02	*PRDM16, TLR4*
fucose metabolic process	2	5.20E-02	*POFUT1, FPGT*
brown fat cell differentiation	2	6.90E-02	*ADRB2, PRDM16*
negative regulation of myeloid cell differentiation	2	8.50E-02	*PRDM16, TLR4*

## DISCUSSION

miRNAs are critical regulators of gene expression and they control many physiologic processes in mammalian cells [5–7]. There are abundant evidences that aberrantly expressed miRNAs can dysregulate otherwise well-controlled cellular RNA networks, thereby enhancing cancer cell development, progression, and metastasis [6–9]. The discovery of aberrantly expressed miRNAs and the resultant changes in RNA networks in cancer cells provide novel molecular explanations for cancer cell progression and metastasis. It is now apparent that dysregulated miRNAs play important roles in BC cell development [16]. Our past miRNA studies of BC cells showed that clustered miRNAs (including *miR-1*/*133a* (targeting *TAGLN2*), *miR-23b*/*27b*/*24-1* (targeting *EGFR*, *MET*, and *FOXM1*), and *miR-195*/*497* (targeting *BIRC5* and *WNT7A*)) act as tumor-suppressive miRNAs through their regulation of several oncogenic genes and pathways [10, 17–19].

Improved technological developments (next generation sequencing) have illuminated the role of miRNA networks in cancer cells. In this study, we examined the expression of *miR-145-5p* and *miR-145 3p* in BC cells because these miRNAs were significantly reduced in cancer cells as determined by deep sequencing. Our data demonstrated that *miR-145-3p* (the passenger-strand from *pre-miR-145*) had anti-tumor effects through targeting of *UHRF1* in BC cells.

Downregulation of *miR-145-5p* (the guide-strand) is frequently observed in many types of cancer, and past studies have established the anti-tumor function of *miR-145-5p* through its regulation of several types of oncogenes in cancer cells [15]. Our group also identified the anti-tumor function of *miR-145-5p* in prostate cancer, renal cell carcinoma, bladder cancer, and esophageal squamous cell carcinoma [20–23]. Importantly, *p53* appears to transcriptionally regulate *miR-145-5p* by interaction with a potential *p53* response element at the *pre-miR-145* promoter region [24]. Moreover, *c-MYC* is directly repressed by *miR-145-5p*, indicating that it acts as a new member of the *p53* regulatory network and contributes to the direct linkage between *p53* and *c-MYC* in human cancer pathways [24]. In contrast to *miR-145-5p*, the functional significance of *miR-145-3p* in cancer cells has been obscure. This is the first report to evaluate the anti-tumor function of *miR-145-3p* in BC cells by gain-of-function assays.

miRNAs are often associated in clusters in the genome, and several studies have focused on the functional role of clustered miRNAs in human cancers [17, 18, 20–23, 25]. In the human genome, 429 human miRNAs have been found to be clustered at 144 sites, with inter-miRNA distances of less than 5,000 base pair (miRBase, release 21). Both *miR-143* and *miR-145-5p* are known to be located close together on human chromosome 5q32, where they form a cluster [26]. Based on our miRNA signatures, *miR-143* and *miR-145-5p* are the most frequently downregulated miRNAs in various types of human cancers [26]. These two miRNAs have been reported as tumor suppressors and studied extensively for their role in oncogenic pathways in several cancers [15]. Our past studies demonstrated that *hexokinase-2* (*HK2*) and *Golgi membrane protein 1* (*GOLM1*) were directly regulated by *miR-143* and *miR-145-5p* in renal carcinoma and prostate cancer, respectively [22, 23].

In this study, we speculated that *miR-145-5p* and *miR-145-3p* worked together to regulate pathways in BC cell progression and metastasis. Our present data showed that *UHRF1* was directly regulated by both *miR-145-5p* and *miR-145-3p* in BC cells. In previous studies of miRNA regulation of *UHRF1* in cancers, *UHRF1* was regulated by *miR-146a*/*146b* in gastric cancer [27], *miR-9* in colorectal cancer [28], and *miR-124* in BC [29]. However, there have been no previous reports about the effects of *miR-145-5p* and *miR-145-3p* on *UHRF1*.

The *UHRF1* gene was first cloned as a transcription factor that binds to the promoter region of the topoisomerase IIα (*TOP2A*) gene and controls its expression levels [30]. UHRF1 is involved in a wide range of physiological and pathological phenomena, including cancer development and metastasis [31]. UHRF1 plays a pivotal role in controlling gene expression through regulating epigenetic mechanisms, including DNA methylation, histone deacetylation, histone methylation, and histone ubiquitination [31]. Overexpression of *UHRF1* occurs in many types of cancer, and aberrantly expressed UHRF1 causes cancer cell activation through hyper-methylation of tumor-suppressor genes such as *BRCA1*, *CDKN2A*, *p73*, and *RASSF1* [32]. Expression of *UHRF1* might be used as a progression marker in cancer [32]. For example, the expression of *UHRF1* in MIBC was greater than in NMIBC, and upregulation was associated with an increased risk of progression after transurethral resection [33]. Our present data showed that knockdown of *UHRF1* significantly induced apoptosis in BC cells and expression levels of the gene correlated with cause specific survival. Our data support the past studies of *UHRF1* in cancer research, suggesting *UHRF1* plays essential roles in BC cell progression and might be a molecular target for BC treatment.

In this study, we identified *UHRF1*-regulated BC pathways by using genome-wide gene expression analysis of *si-UHRF1*-transfected cells. Our expression data showed that *UHRF1* and *TOP2A* were significantly reduced by *si-UHRF1* transfection, indicating the usefulness of the present analytic approach. Our data showed that several anti-apoptosis genes and pro-proliferation genes were involved in pathways downstream of *UHRF1*, such as *BIRC5* and *CENPF*. *BIRC5* is a member of the inhibitor of apoptosis (IAP) family preferentially expressed by many cancers, including BC [10], and its mediated cellular networks are essential for cancer cell proliferation and viability [34]. *CENPF* is a master regulator of prostate cancer malignancy. Together, *FOXM1* and *CENPF* regulate target gene expression and activation in cancer cells [35, 36]. The identification of these novel molecular pathways and targets mediated by the *miR-145-5p*/*145-3p*/*UHRF1* axis may lead to a better understanding of BC cell progression and metastasis.

In conclusion, downregulation of dual-strand *miR- 145-5p* and *miR-145-3p* was validated in BC clinical specimens, and these miRNAs were shown to function as tumor suppressors in BC cells. To the best of our knowledge, this is the first report demonstrating that tumor suppressive *miR-145-5p* and *miR-145-3p* directly targeted *UHRF1*. Moreover, *UHRF1* was upregulated in BC clinical specimens and contributed to anti-apoptotic effects through its regulation of several oncogenic genes. Expression of *UHRF1* might be a useful prognostic marker for survival of BC patients. The identification of novel molecular pathways and targets regulated by the *miR-145-5p*/*miR-145-3p*/*UHRF1* axis may lead to a better understanding of BC progression and aggressiveness.

## MATERIALS AND METHODS

### Clinical specimens and cell lines

Clinical tissue specimens were collected from BC patients (*n* = 69) who had undergone transurethral resection of their bladder tumors (TURBT, *n* = 59) or cystectomy (*n* = 10) at Kagoshima University Hospital between 2003 and 2013. NBE (*n* = 12) were derived from patients with noncancerous disease. The specimens were staged according to the American Joint Committee on Cancer-Union Internationale Contre le Cancer tumor-node-metastasis (TNM) classification and histologically graded [37]. Our study was approved by the Bioethics Committee of Kagoshima University; written prior informed consent and approval were obtained from all patients. Patient details and clinicopathological characteristics are listed in Table 6.

**Table 6:** Characteristic of patients

Bladder cancer (BC)			
Total number	69		
Median age (range)	73	(40–94)	years
Gender			
Male	53	76.8%	
Female	16	23.2%	
Tumor grade			
Low grade	45	65.2%	
High grade	22	31.9%	
Unknown	2	2.9%	
T stage			
Tis	2	2.9%	
Ta	7	10.1%	
T1	25	36.2%	
T2	27	39.1%	
T3	4	5.8%	
T4	4	5.8%	
N stage			
N0	40	58.0%	
N1	8	11.6%	
Unknown	21	30.4%	
M stage			
M0	58	84.1%	
M1	5	7.2%	
Unknown	6	8.7%	
Operation method			
TURBT	59	85.5%	
Cystectomy	10	14.5%	
**Normal bladder epithelium**			
Total number	12		
Median age (range)	61	(47–72)	years

Abbreviation: TURBT = transurethral resection of bladder tumor

We used two human BC cell lines: T24, which was invasive and obtained from the American Type Culture Collection; and BOY, which was established in our laboratory from an Asian male patient, 66 years old, who was diagnosed with stage III BC and lung metastasis [38, 39]. These cell lines were maintained in minimum essential medium supplemented with 10% fetal bovine serum in a humidified atmosphere of 5% CO_2_ and 95% air at 37°C.

### Tissue collection and RNA extraction

Tissues were immersed in RNAlater (Thermo Fisher Scientific; Waltham, MA, USA) and stored at −20°C until RNA extraction was conducted. Total RNA, including miRNA, was extracted using the mirVana^™^ miRNA isolation kit (Thermo Fisher Scientific) following the manufacturer's protocol. The integrity of the RNA was checked with an RNA 6000 Nano Assay kit and a 2100 Bioanalyzer (Agilent Technologies, Santa Clara, CA, USA) following the manufacturer's protocol.

### Quantitative real-time reverse transcription polymerase chain reaction (qRT-PCR)

The procedure for qRT-PCR quantification was described previously [40, 41]. Stem-loop RT-PCR (TaqMan MicroRNA Assays; product ID: 002278 for *miR-145-5p* and product ID: 002149 for *miR-145-3p*; Thermo Fisher Scientific) was used to quantify miRNAs according to previously published conditions [40–42]. TaqMan probes and primers for *UHRF1* (product ID: Hs 01086727_m1; Thermo Fisher Scientific) were assay-on-demand gene expression products. We used human *GUSB* (product ID: Hs99999908_m1; Thermo Fisher Scientific) and *RNU48* (product ID: 001006; Thermo Fisher Scientific), respectively, as internal controls.

### Transfections with miRNA mimic and small interfering RNA (siRNA) into BC cell lines

Mature miRNA molecules, Pre-miR^™^ miRNA precursors (*hsa-miR-145-5p*; product ID: PM11480, *hsa-miR-145-3p*; product ID: PM13036, and negative control miRNA; product ID: AM 17111; Thermo Fisher Scientific) were used in the gain-of-function experiments, whereas *UHRF1* siRNA (product ID: HSS120939 and HSS179006; Thermo Fisher Scientific) and negative control siRNA (product ID: D-001810-10; Thermo Fisher Scientific) were used in the loss-of-function experiments. The transfection procedures and transfection efficiencies of miRNA in T24 and BOY cells were reported previously [40–42].

### Cell proliferation, migration, and invasion assays

To investigate the functional significance of the *miR-145-5p*, *miR-145-3p*, and *UHRF1*, we performed cell proliferation, migration, and invasion assays using T24 and BOY cells. The experimental procedures were performed as described in our previous studies [40–42].

### Apoptosis assays

BC cell lines were transiently transfected with reagent only (mock), miR-control, *miR-145-5p*, *miR- 145- 3p*, siRNA-control, or *si-UHRF1* at 10 nM in 6 well tissue culture plates, as described previously [14, 17–19]. Cells were harvested by trypsinization 72 hours after transfection and washed in cold phosphate-buffered saline. For apoptosis assays, double staining with FITC-Annexin V and propidium iodide was carried out using a FITC Annexin V Apoptosis Detection Kit (BD Biosciences, Bedford, MA, USA) according to the manufacturer's recommendations and analysed within 1 hour by flow cytometry (CyAn ADP analyzer; Beckman Coulter, Brea, CA, USA). Cells were identified as viable cells, dead cells, early apoptotic cells, and apoptotic cells using Summit 4.3 software (Beckman Coulter), and the percentages of early apoptotic and apoptotic cells from each experiment were then compared. As a positive control, we used 2 μg/mL cycloheximide.

### Cell cycle assays

For the cell cycle analyses, cells were stained with PI using the Cycletest PLUS DNA Reagent Kit (BD Biosciences) following the protocol and analyzed by CyAn ADP analyzer (Beckman Coulter). The percentages of the cells in the G0/G1, S, and G2/M phases were determined and compared. Experiments were performed in triplicate.

### Western blot analyses

Immunoblotting was performed with rabbit anti-UHRF1 antibodies (1:500, PA5-29884; Thermo Fisher Scientific), anti-PARP antibodies (1:500 #9542; Cell Signaling Technology; Danvers, MA, USA), anti-cleaved PARP antibodies (1:500 #5625; Cell Signaling Technology), and anti-GAPDH antibodies (1:10000 MAB374; Chemicon, Temecula, CA, USA). Specific complexes were visualized with an echochemiluminescence detection system (GE Healthcare, Little Chalfont, UK).

### Immunohistochemistry

A tissue microarray of 68 urothelial cancers and 20 normal bladder tissues was obtained from US Biomax, Inc. (Rockville, MD, USA) (product ID: BL1002). Detailed information on all tumor specimens can be found at http://www.biomax.us/index.php. The tissue microarray was immunostained following the manufacturer's protocol with an Ultra Vision Detection System (Thermo Scientific). The primary rabbit polyclonal antibodies against UHRF1 (PA5-29884; Thermo Fisher Scientific) were diluted 1:300. Immunostaining was evaluated according to a scoring method as described previously [17].

### Genome-wide gene expression and *in silico* analyses for the identification of genes regulated by *miR-145-5p* and *miR-145-3p*

To further investigate the specific genes affected by *miR-145-5p* and *miR-145-3p*, we performed a combination of *in silico* and genome-wide gene expression analyses. We attempted to identify target genes using a BC cell line transfected with these miRNAs. A Sure Print G3 Human GE 8 × 60K Microarray (Agilent Technologies) was used for expression profiling of *miR-145-5p* and *miR-145-3p* transfectants. The microarray data were deposited into GEO (http://www.ncbi.nlm.nih.gov/geo/) and were assigned GEO accession number GSE66498. Next, we selected putative miRNA target genes using the microRNA.org database (August, 2010 release, http://www.microrna.org). Finally, to identify upregulated genes in BC, we analyzed publicly available gene expression data sets in GEO (accession numbers: GSE11783, GSE31684). The data were normalized and analyzed with Gene Spring software (Agilent Technologies) as described previously [22, 23, 40–42]. The strategy for investigation of the target genes is shown in Figure 3.

### Plasmid construction and dual luciferase reporter assays

Partial wild-type sequences of the 3′ UTR of *UHRF1* or those with a deleted *miR-145-5p* and *miR- 145- 3p* target site (positions 1,179–1,198 of *UHRF1* 3′ UTR for *miR- 145-5p*, and positions 287–292 of *UHRF1* 3′ UTR for *miR-145-3p*) were inserted between the XhoI and PmeI restriction sites in the 3′ UTR of the *hRluc* gene in the psiCHECK-2 vector (C8021; Promega, Madison, WI, USA). T24 and BOY cell lines were transfected with 50 ng of the vector and 10 nM *miR-145-5p* or *miR-145-3p* using Lipofectamine 2000 (Thermo Fisher Scientific) and Opti-MEM (Thermo Fisher Scientific). The activities of firefly and *Renilla* luciferases in cell lysates were determined with a dual luciferase reporter assay system according to the manufacturer's protocol (E1960; Promega). Normalized data were calculated as the ratio of *Renilla*/firefly luciferase activities.

### Identification of downstream targets regulated by *UHRF1* in BC

To investigate molecular targets regulated by *UHRF1* in BC cells, we carried out gene expression analyses using *si-UHRF1*-transfected BC cell lines. Microarray data were used for expression profiling of *si-UHRF1* transfectants. The microarray data were deposited into GEO (accession number: GSE77790). We analyzed common down or upregulated genes using the GEO dataset. The flow chart outlining the investigation of *UHRF1* downstream genes is shown in Figure 10A and 10B.

### Statistical analysis

Relationships among two or three variables and numerical values were analysed using the Mann-Whitney *U* test or Bonferroni-adjusted Mann-Whitney *U* test. Spearman's rank test was used to evaluate the correlation among the expressions of *miR-145-5p*, *miR-145-3p*, and *UHRF1*. We estimated cause specific survival of 57 BC patients by using the Kaplan-Meier method. Among the 69 BC patients, 12 died of other causes. Therefore, we analyzed cause specific survival of 57 BC patients. Patients were divided into two groups according to the median value of *UHRF1* expression, and the differences between the two groups were evaluated by the log-rank tests. We used Expert Stat View software, version 5.0 (SAS Institute Inc., Cary, NC, USA), for these analyses.

## SUPPLEMENTARY MATERIALS FIGURES


