# Supplementary material for: Regulation of UHRF1 by dual-strand tumor-suppressor microRNA-145 (miR-145-5p and miR-145-3p): inhibition of bladder cancer cell aggressiveness
Source: Oncotarget. 2016 Apr 9;7(19):28460–87. doi: 10.18632/oncotarget.8668 (PMC5053739; doi:10.18632/oncotarget.8668)
Supplement: Supplementary file 1 [file oncotarget-07-28460-s001.pdf]

Regulation of *UHRF1* by dual-strand tumor-suppressor *microRNA-145* (*miR-145-5p* and *miR-145-3p*): inhibition of bladder cancer cell aggressiveness

Supplementary Materials

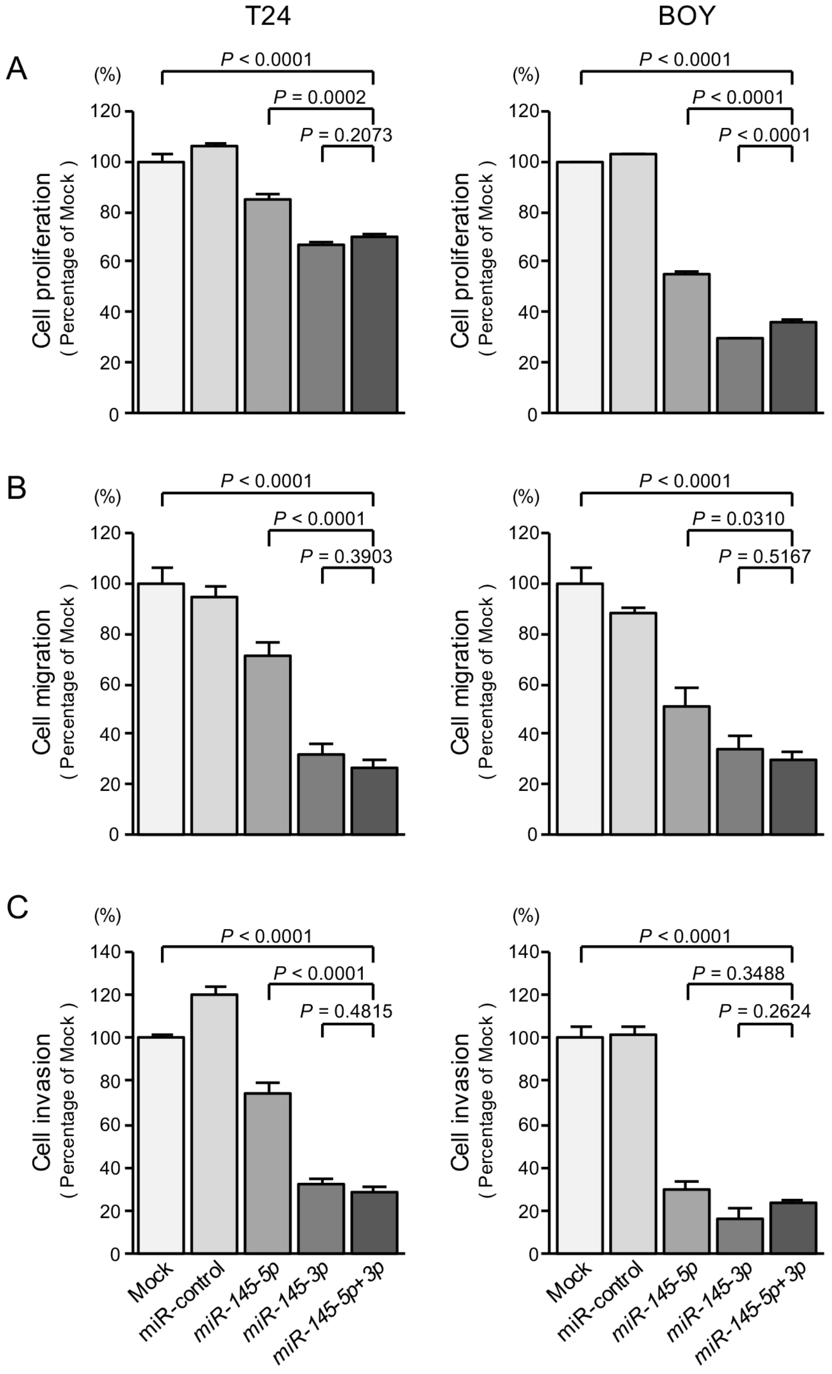

Supplementary Figure S1: Effects of co-transfection of *miR-145-5p* and m*iR-145-3p* on proliferation, migration and invasion assays. Co-transfection of *miR-145-5p* (10 nM) and *miR-145-3p* (10 nM) did not show synergistic effects on cancer cell aggressiveness compared with *miR-145-5p* or *miR-145-3p* transfection in BC cells.

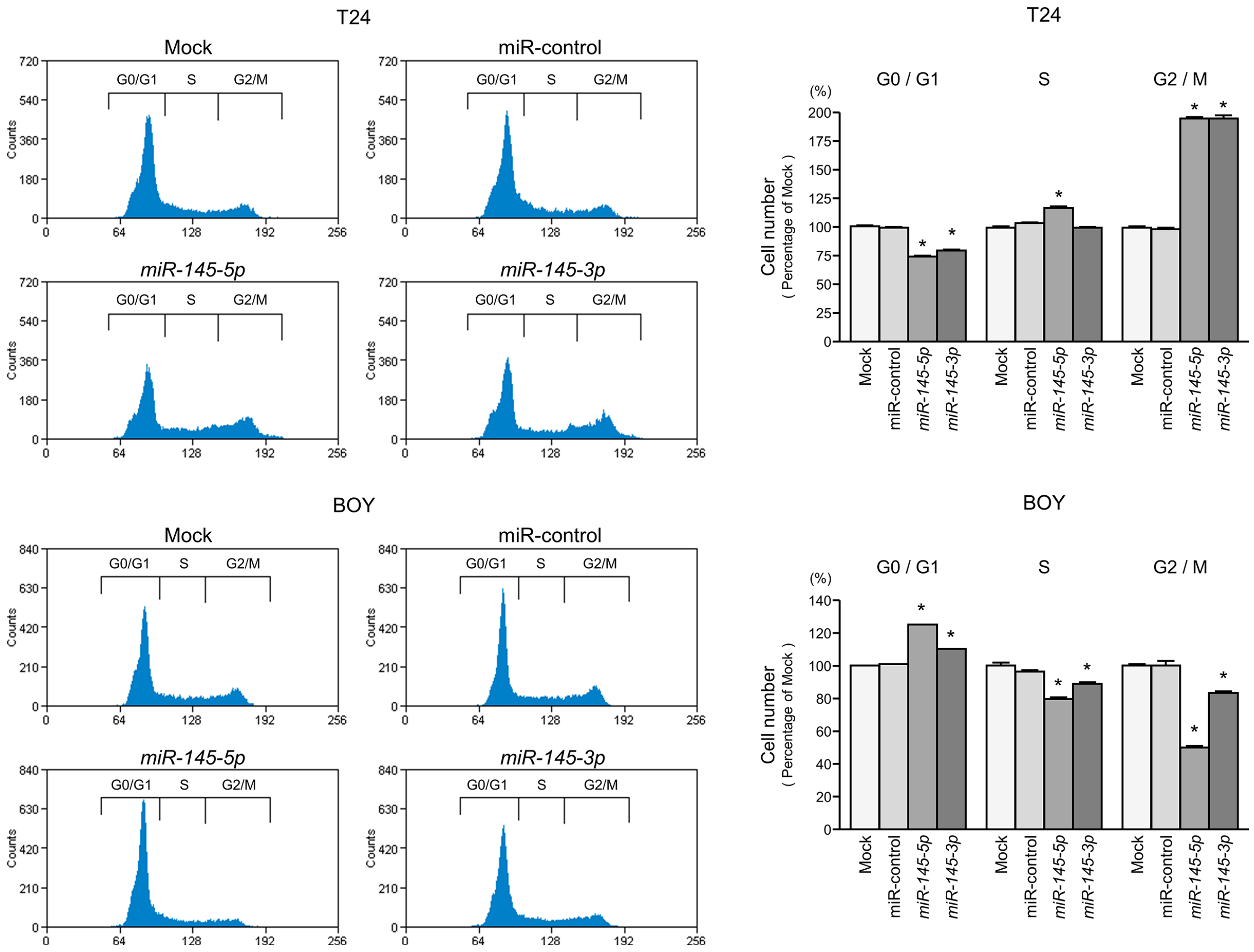

Supplementary Figure S2: Cell cycle assays by *miR-145-5p* and *miR-145-3p* transfectants in BC cells. Flow cytometric analysis of cell cycle phase distribution in mock, miR-control, or *miR-145-5p* and *miR-145-3p* transfectants. The bar charts represent the percentage of mock in G0/G1, S, and G2/M phases. *$P < 0.0001$.
